# Mechanistic insights into SteAB regulation of cell wall hydrolase RipA in *Mycobacterium tuberculosis*

**DOI:** 10.1128/mbio.03700-25

**Published:** 2026-01-26

**Authors:** Giacomo Carloni, Quentin Gaday, Daniela Megrian, Julienne Petit, Mariano Martinez, Adrià Sogues, Mathilde Ben Assaya, Marcell Kakonyi, Ahmed Haouz, Pedro M. Alzari, Anne Marie Wehenkel

**Affiliations:** 1 Institut Pasteur, Université Paris Cité, CNRS UMR 3528, Bacterial Cell Cycle Mechanisms Unit, Paris, France; 2 Institut Pasteur, Université Paris Cité, CNRS UMR 3528, Structural Microbiology Unit, Paris, France; 3 Institut Pasteur de Montevideo, Bioinformatics Unit, Montevideo, Uruguay; 4 Institut Pasteur, Plate-forme de cristallographie – C2RT, CNRS UMR 3528, Université Paris Cité, Paris, France; Weill Cornell Medicine, New York, New York, USA

**Keywords:** bacterial cell division, peptidoglycan remodeling, *Mycobacterium tuberculosis*, structural biology

## Abstract

D,L-endopeptidase RipA is the major peptidoglycan (PG) hydrolase required for cell separation in *Mycobacterium tuberculosis* (*Mtb*), as RipA defects severely hinder cell division and increase antibiotic vulnerability. Despite extensive studies, the mechanisms governing *Mtb* RipA regulation remain controversial and poorly understood. Here, we report an integrative structural and functional analysis of the *Mtb* SteAB system, a regulatory transmembrane septal complex encoded by adjacent genes *Rv1697* (*steA*) and *Rv1698/mctB* (*steB*) that are conserved across *Mycobacteriales*. The separate crystal structures of the cytoplasmic core of *Mt*SteA and the periplasmic core of *Mt*SteB in complex with the RipA coiled-coil domain (RipA_CC_), along with biochemical evidence that these proteins form a stable transmembrane physical complex, define the function of the SteAB complex as a regulator of RipA-mediated cell separation in *Mtb*, arguing against a previously proposed role of MctB (Rv1698) as a putative mycobacterial outer membrane copper transporter. Our structural findings showed that, upon *Mt*SteB binding, RipA_CC_ is oriented perpendicular to the membrane, bringing its endopeptidase catalytic domain in physical contact with the PG layer, while the homodimeric structural core of SteA revealed a conserved functional pocket similar to the phosphonucleotide-binding site of thiamine pyrophosphokinase. These data, coupled with the *in vivo* phenotypic analysis of a *steAB* knockout mutant of *Cglu*, support a model in which the SteAB heterotetramer orchestrates the productive positioning of RipA leading to PG hydrolysis activation. These findings shed new light on the regulation of mycobacterial cell wall remodeling, with implications for understanding *Mtb* pathogenesis and identifying novel antimicrobial targets.

**IMPORTANCE:**

Peptidoglycan (PG) is a major component of the bacterial cell wall. A flexible but strong PG mesh encloses the cell, conferring mechanical resistance and preventing cell lysis. This PG mesh is continually remodeled during the bacterial life by the coordinated action of tightly regulated PG-hydrolases and synthetases. Here, we report the structural characterization of the *M. tuberculosis* SteAB system, which regulates the action of the major enzyme responsible for disassembling the PG mesh to allow daughter cell separation at the end of cell division. The proposed model for the septal control of PG hydrolysis illustrates how the transmembrane SteAB complex can promote enzyme activation and provides structural information that may help target the activation mechanism for antibiotic development.

## INTRODUCTION

Peptidoglycan (PG), the main component of the bacterial cell wall, consists of glycan strands of two alternating sugar molecules, N-acetylglucosamine (NAG) and N-acetylmuramic acid (NAM), cross-linked by short pentapeptide stems. This cage-like polymer surrounds the plasma membrane, confers cell shape and protection against osmotic disruption, and is continually remodeled during the cell cycle through the coordinated action of different PG hydrolases and synthetases (1). In polar-growing bacteria, such as *Mycobacteriales*, which do not undergo septal constriction during cytokinesis, the PG mesh forms a continuous mechanical linkage between the progeny cells at the nascent division plane. At the end of cell division, this linkage must be released to allow for daughter cell separation (V-snapping) (2). This crucial task is performed by an array of PG hydrolases, whose tight regulation ensures that cell division occurs in a timely and organized manner, preventing uncontrolled cell wall degradation that can lead to morphological defects or bacterial lysis. These defective cell division phenotypes are often accompanied by increased membrane permeability and antibiotic susceptibility, making PG hydrolases attractive targets for the development of novel antimicrobial agents.

In the human pathogen *Mycobacterium tuberculosis* (*Mtb*), D-L endopeptidase RipA (Rv1477) is the major PG hydrolase involved in cell separation (3–5). Although there are other genes encoding PG hydrolases in *Mtb*, RipA is the only endopeptidase whose depletion induces severe morphological defects and *in vivo* reduced infectivity, emphasizing its importance for proper cell separation and integrity. RipA is a member of a conserved clade of the NlpC/P60 enzyme superfamily that cleaves stem peptide bridges within the PG mesh and has been shown to be important for cell separation in other *Mycobacteriales* species (2, 6–8). Despite its essential role in *Mtb* cell division, limited and controversial information is available regarding the underlying regulatory mechanism(s) of this process. RipA interacts *in vivo* with other cell division proteins, notably the penicillin-binding protein PBP1 and resuscitation-promoting factor RpfB (9, 10), which may be involved in enzyme regulation through protein-protein interactions. Moreover, truncated RipA species were found in cell wall compartments and culture filtrates, suggesting that RipA is proteolytically processed *in vivo* (4), and the protease MarP was reported to hydrolyze RipA during acid stress (11).

More recently, it has been shown that the periplasmic membrane-associated protein SteB binds to and activates full-length RipA in *Corynebacterium glutamicum (Cglu*) by dissociating the intramolecular complex between the catalytic and the EnvC-like coiled-coil (CC) domains (12). SteB is part of a septal transmembrane complex with the cytosolic membrane protein SteA, and inactivation of either *steA* or *steB* (adjacent genes in the same operon) phenocopies RipA inactivation in terms of ethambutol hypersensitivity and cell wall defects (13). These findings demonstrated that the SteAB complex is part of a regulatory system for cell wall degradation mediated by RipA (12). However, the underlying molecular mechanisms remain unclear. Although the *steAB* operon is conserved in *Mycobacteriales*, the SteB homolog in *Mtb* (Rv1698) has previously been described as a putative channel-forming protein involved in copper transport (14, 15) and therefore renamed MctB (for Mycobacterial copper transport protein B). The *Mtb* SteA homolog (Rv1697) is an uncharacterized hypothetical protein annotated as a thiamin pyrophosphokinase that is essential for the *in vitro* growth of *Mtb* H37Rv (16–19). For clarity, we will refer to these proteins, respectively, as *Mt*SteA (Rv1697) and *Mt*SteB (Rv1698) in the rest of this manuscript.

Here, we report an integrative structural analysis of the SteAB system in *Mtb*. The separate crystal structures of the *Mt*SteA homodimer and the *Mt*SteB homodimer in complex with the CC domain of RipA, along with evidence that these proteins form a stable transmembrane physical complex, redefine the role of the SteAB complex as a regulator of RipA in Mtb, dismissing a direct involvement of *Mt*SteB in copper transport as an outer-membrane channel protein. Our structural findings, combined with the phenotypic analysis of a SteAB-deficient *Cglu* mutant strain, put forward a model where the formation of the transmembrane SteAB heterotetramer promotes the effective positioning of RipA to initiate PG hydrolysis in the periplasm, possibly triggered by ligand binding to *Mt*SteA in the cytoplasm. Elucidating the intricacies of RipA function enhances our understanding of *Mtb* pathogenesis and opens new avenues for the design of innovative strategies to combat tuberculosis.

## RESULTS

### The structures of *M. tuberculosis* SteB, alone and in complex with the RipA coiled-coil domain

**Fig 1:**
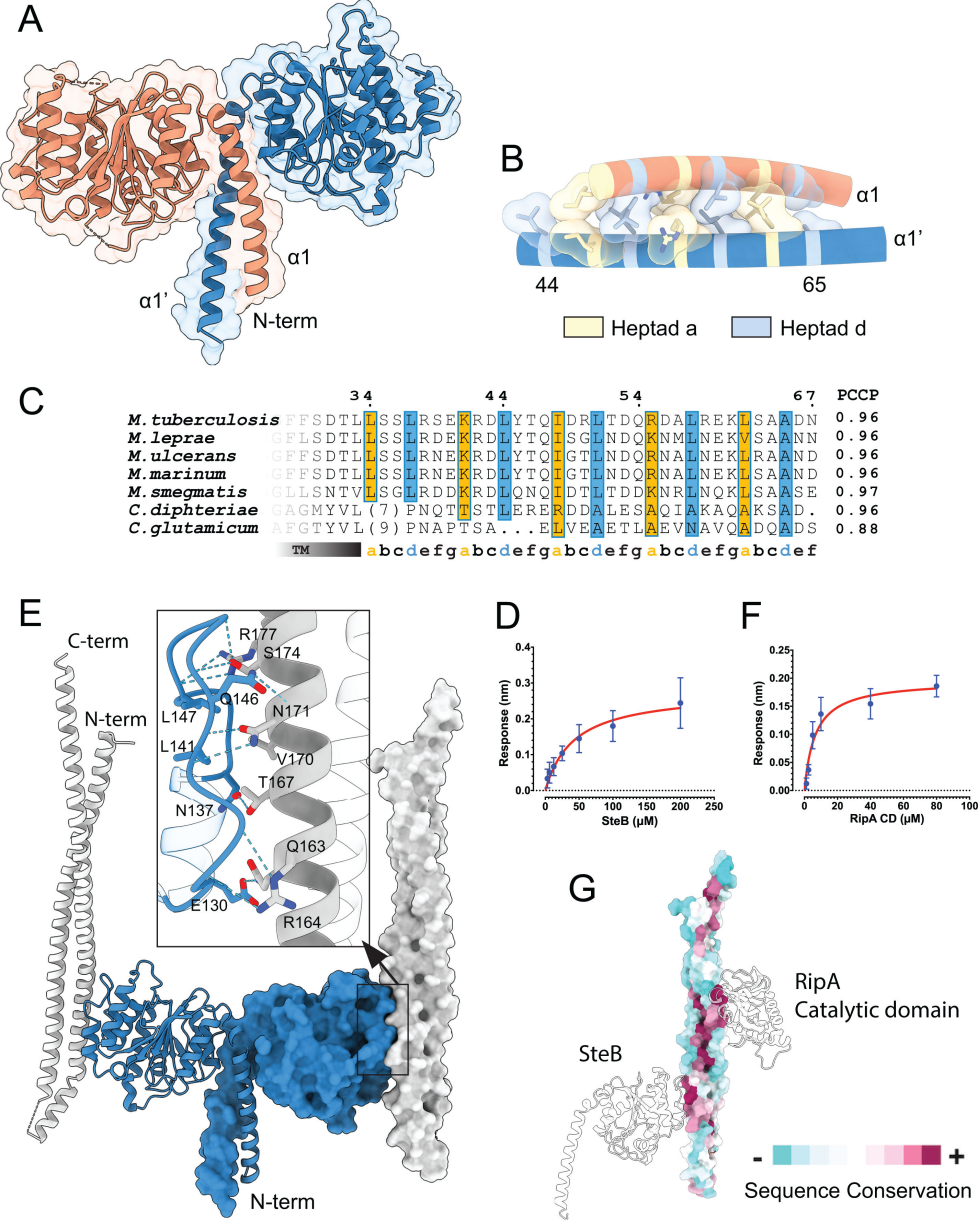
Overall structure of *Mt*SteB. (**A**) Cartoon representation of the *Mt*SteB_△TM_ homodimer, with each monomer in pink and blue, respectively. (**B**) Detailed view of the N-terminal coiled-coil (CC), showing the residue side-chains at the *a* and *d* positions of the heptad repeats. (**C**) Alignment of the SteB CC region from selected species of *Mycobacteriales*. The TM region and the (*a–g*) heptad repeat positions are shown below the alignment, with the *a* and *d* positions shown in orange and blue, respectively. PCCP indicates the probability of parallel CC formation (20). (**D**) BLI binding curves of immobilized *Mt*RipA_CC_ against *Mt*SteB. The response (vertical axis) is measured as the wavelength shift in nanometers (nm). (**E**) Overall view of the crystal structure of the *Mt*SteB_△TM_ homodimer (blue) in complex with two *Mt*RipA_CC_ molecules (light gray). One of the SteB-RipA complexes is shown in cartoon representation and the other in surface representation. The SteB dimer is shown in a similar orientation as in panel **A**. The inset shows a ribbon representation of the interface, in which residues involved in hydrogen bonding interactions are labeled. (**F**) BLI binding curves of immobilized *Mt*RipA_CC_ against *Mt*RipA_CAT_. (**G**) The analysis of residue conservation (ConSurf) in all *Mycobacteriales* identifies two non-overlapping regions in the N-terminal CC of RipA (shown here in the same orientation as in panel **E**) that interact with the C-terminal catalytic domain and the activator protein SteB, respectively.

*Mt*SteB is a 33 kDa membrane-bound protein (314 amino acids) with a single N-terminal transmembrane (TM) segment. For structural studies, we produced the soluble protein devoid of its TM domain (*Mt*SteB_△TM_, residues 38–314) and determined its crystal structure at 2 Å resolution. The protein is a homodimer with a central CC dimerization domain (residues 41–76 from each protomer), surrounded on either side by the respective C-terminal globular cores (residues 77–314) (Fig. 1A). The monomeric core, which displayed a (β/α) topology (Fig. S1A), is similar to that of the homologous SteB from *Cglu* ((12), pdb code 8AU6), with a root-mean-square-deviation (RMSD) of 1.019 Å for 168 Cα equivalent positions (Fig. S1B). A major difference, however, is that *Cg*SteB crystallized as a monomer, whereas *Mt*SteB is a homodimer. The dimeric conformation is mediated by the intermolecular parallel CC formed between the N-terminal α-helices of each protomer (Fig. 1B). The CC was further stabilized by interactions of the helix tip from one protomer with the C-terminus of the second protomer (Fig. S1C). In *Cg*SteB, this C-terminal region (residues 296–314) was missing from the construct (12), possibly accounting for its crystallization as a monomer. Close inspection of the residues involved in CC formation revealed that the heptad repeat pattern is conserved in SteB homologs from other *Mycobacteriales* (Fig. 1C). Although the heptad repeats in the *Cglu* protein show slightly lower conservation, gel filtration experiments indicate that *Cg*SteB exists as a dimer in solution (Fig. S2A). Furthermore, the AlphaFold (AF) model of *Cg*SteB (Fig. S2B) predicts the same CC-mediated dimerization mode as seen in *Mt*SteB crystals, thereby supporting the SteB homodimer as the putative functional unit. In the full-length protein, the TM helix immediately precedes the CC helix (Fig. 1C) and may thus play a role in stabilizing (or modifying) the CC domain structure, suggesting a possible mechanism for conformational signal transduction.

Given the structural similarity between the SteB homologs from *Mtb* and *Cglu*, it is likely that *Mt*SteB, like *Cg*SteB, plays a direct regulatory role for RipA (12), rather than the initially proposed role in copper transport (15). Supporting this hypothesis, we observed that *Mt*SteB_△TM_ interacted with the CC domain (residues 40–240) of RipA (*Mt*RipA_CC_) with an apparent dissociation constant *Kd* of 42.6 ± 4.4 μM (Fig. 1D; Fig. S3), as determined by bio-layer interferometry (BLI). Furthermore, we crystallized the *Mt*SteB_△TM_ *– Mt*RipA_CC_ complex and solved its 3D structure at 2.2 Å resolution (Table S1). The structure revealed a 2:2 heterotetramer (Fig. 1E), where the *Mt*SteB homodimer is identical to that observed in the *apo* structure. *Mt*RipA_CC_ folds into an antiparallel helical hairpin, with helices α1 (residues 46–120) and α2 (residues 138–238). The two *Mt*RipA_CC_ molecules in the complex aligned parallel to each other and were roughly perpendicular to the membrane plane. Protein-protein association buries a total surface of 690 Å^2^ and is mediated by both an extended intermolecular hydrogen bonding network involving several residues from *Mt*SteB (Glu130, Leu141, Gln146, Gly144, Ser145, Leu147, and Lys150) and *Mt*RipA (Gln163, Arg164, Thr167, Asn171, Ser174, and Arg177), as well as hydrophobic interactions between helix α2 of *Mt*RipA_CC_ and the loop connecting helices α3–α4 (residues 139–153) of *Mt*SteB (Fig. 1E inset). Interestingly, the α3–α4 loop, which is well defined in the structure of the complex, was disordered in the *apo Mt*SteB structure and was not visible in the electron density map (Fig. S4), indicating an induced-fit mechanism for RipA recruitment.

### *Mt*RipA is autoinhibited *via* its N-terminal CC domain

The interface of the SteB-RipA complex in *Mtb* described above is similar to that previously predicted for the homologous complex in *Cglu*, which was validated by site-directed mutagenesis (12). While this similarity suggests a conserved autoinhibition/activation mechanism of RipA, the available crystallographic structures of full-length or truncated forms of RipA appear to indicate a different autoinhibition mechanism in these two species: crystals of full-length *Cg*RipA revealed the N-terminal CC domain bound to and blocking the catalytic site (12), whereas two independent crystal structures of a truncated form of *Mt*RipA lacking the N-terminal CC domain (21, 22) showed the active site blocked by the linker region immediately preceding the catalytic domain (Fig. S5A). These structures are consistent with the hypothesis that *Mt*RipA is a zymogen requiring proteolytic processing (4, 11) and suggest that the N-terminal CC domain of RipA may have a different function (23). To investigate this apparent discrepancy, we predicted structural models of truncated and full-length *Mt*RipA forms using template-free AlphaFold (AF). In the full-length protein, the active site was predicted to bind the N-terminal CC domain to form a complex like that seen for *Cg*RipA (12), while the active site interactions with the linker region were only predicted for the constructs lacking the CC domain (Fig. S5B), suggesting that the previous crystallographic observations of *Mt*RipA (21, 22) were a consequence of protein truncation. To experimentally validate this interaction, we produced separate constructs for *Mt*RipA_CC_ (residues 40–240) and *Mt*RipA_CAT_ (residues 261–472) and measured their binding affinity using BLI. The apparent *Kd* value obtained (6.8 ± 1.4 μM, Fig. 1F; Fig. S5C) strongly supports the CC-mediated autoinhibitory model. In summary, the findings above support a conserved RipA regulation mechanism within *Mycobacteriales* and reveal that the *Mt*RipA CC domain comprises two distinct, highly conserved regions (Fig. 1G) that are used separately for autoinhibition (binding to the catalytic domain) and for activation (binding to *Mt*SteB).

### Structural characterization of SteA

**Fig 2:**
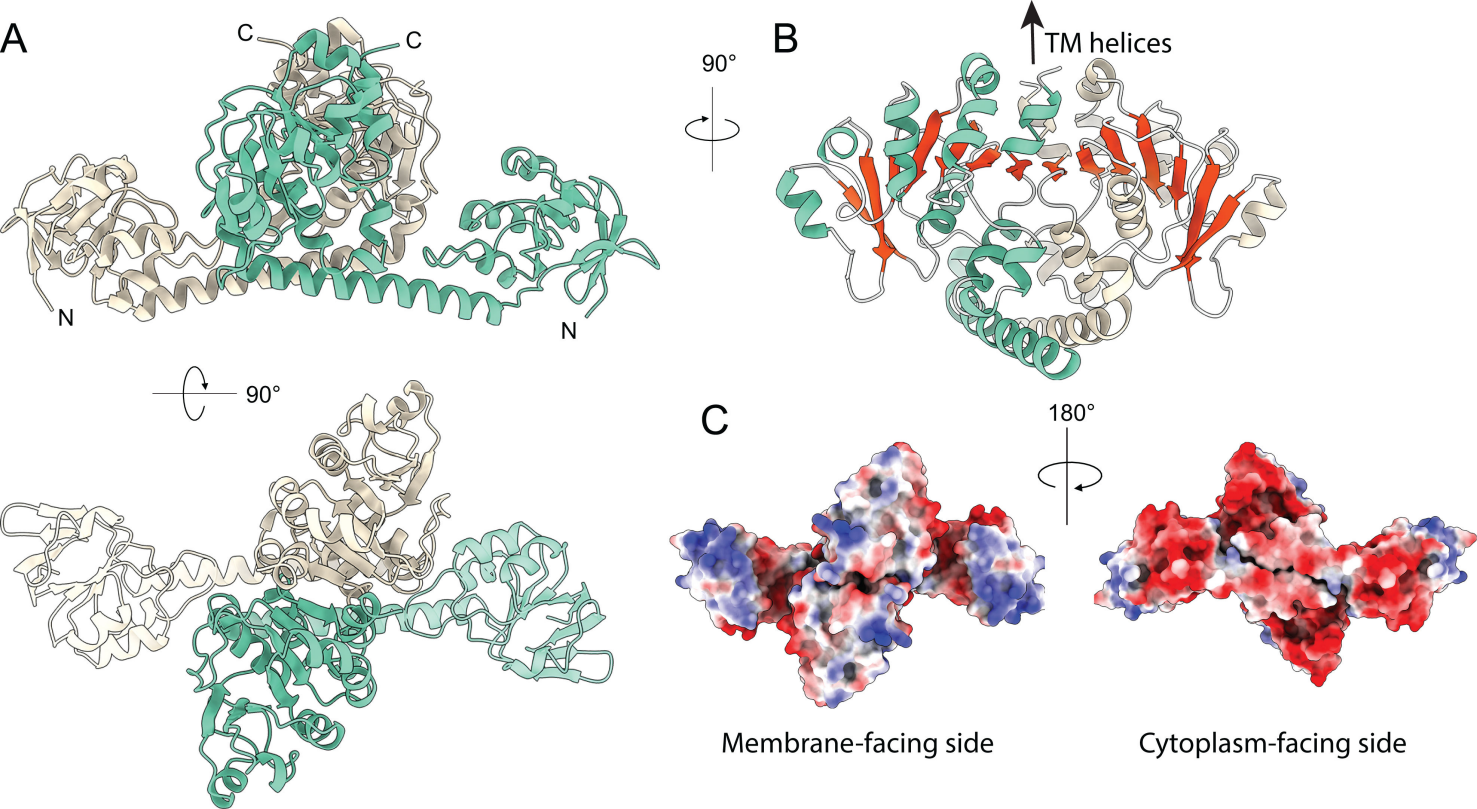
Structure of *Mt*SteA. (**A**) Side and top views of the *Mt*SteA_△TM_ homodimer, with the monomers shown in wheat and green, respectively. (**B**) Protein dimerization results in an intermolecular 14-stranded β-sheet (shown in red) formed by the two C-terminal domains. (**C**) Molecular surface of *Mt*SteA_△TM_ colored by electrostatic charges.

*Mt*SteA is a 43 kDa protein (393 amino acid residues) that consists of an N-terminal cytoplasmic core, followed by a TM helix (residues 344–366) and a C-terminal amphipathic helix exposed on the periplasmic side (residues 371–393). For structural studies, we produced a truncated soluble construct (residues 12–344, *Mt*SteA_△TM_) that forms a dimer in solution (Fig. S6A) and determined its crystal structure at 2.2 Å resolution (Table S1). The *Mt*SteA_△TM_ homodimer exhibits an overall ‘paper boat’ shape (Fig. 2A), in which the monomers fold into a central C-terminal dimerization domain (the sail) connected through a long (31 residues) α-helix (the hull) to the distal N-terminal globular domains. Protein dimerization buries a largely hydrophobic surface of 2070 Å^2^ from the central linker helix to the C-terminal domain of each monomer (accounting for 13% of the total surface area) and is stabilized by several intermolecular hydrogen bonds and two salt bridges involving residues Arg72 and Asp166 from each protomer.

The N-terminal globular domain (residues 12–127) consists of an external four-stranded antiparallel β-sheet orthogonally packed against a four-stranded parallel β-sheet, covered in turn by three helices (Fig. 2A; Fig. S6B). As revealed by a DALI search (24), this three-layer (β/β/⍺) fold resembles that of the swiveling phosphohistidine domain of phosphoenolpyruvate-transferring enzymes (IPR036637) (Fig. S7), although the catalytic histidine is missing in SteA. This structural domain makes only a few intramolecular contacts with the central *Mt*SteA protein core and displays a high intrinsic flexibility. This was confirmed by the crystal structure of the closely similar SteA homolog from *Cglu* (*Cg*SteA), which was determined at 2.05 Å resolution (Table S1) and contained eight independent molecules in the asymmetric unit. In all molecules, the N-terminal domain exhibited high B factors (Fig. S8A), and the overall superposition of the *Cg*SteA and *Mt*SteA monomer structures revealed a wide range of movement of the N-terminal domain (Fig. S8B and Movie S1).

The C-terminal domain of *Mt*SteA (residues 161–342) is responsible for dimerization and forms the central core of the homodimer (Fig. 2A). The domain displayed an (⍺/β)_7_ secondary structure topology (Fig. S6B) in which the parallel β-sheet extends, upon dimerization, into a 14-stranded twisted β-sheet flanked by α-helices on both sides (Fig. 2B). At the C-terminus of the dimeric cytoplasmic core, the last α-helices from each protomer immediately preceding the TM helices run parallel to and interact with each other, defining the orientation of the protein with respect to the membrane. Consistently, the Coulombic electrostatic potential of the protein surface reveals a positively charged membrane-proximal surface (Fig. 2C). This surface includes an array of basic residues (Arg/Lys) that are highly conserved in SteA homologs from *Mycobacteriales* and could interact with negatively charged membrane phospholipids.

### A conserved ligand-binding pocket in SteA

**Fig 3:**
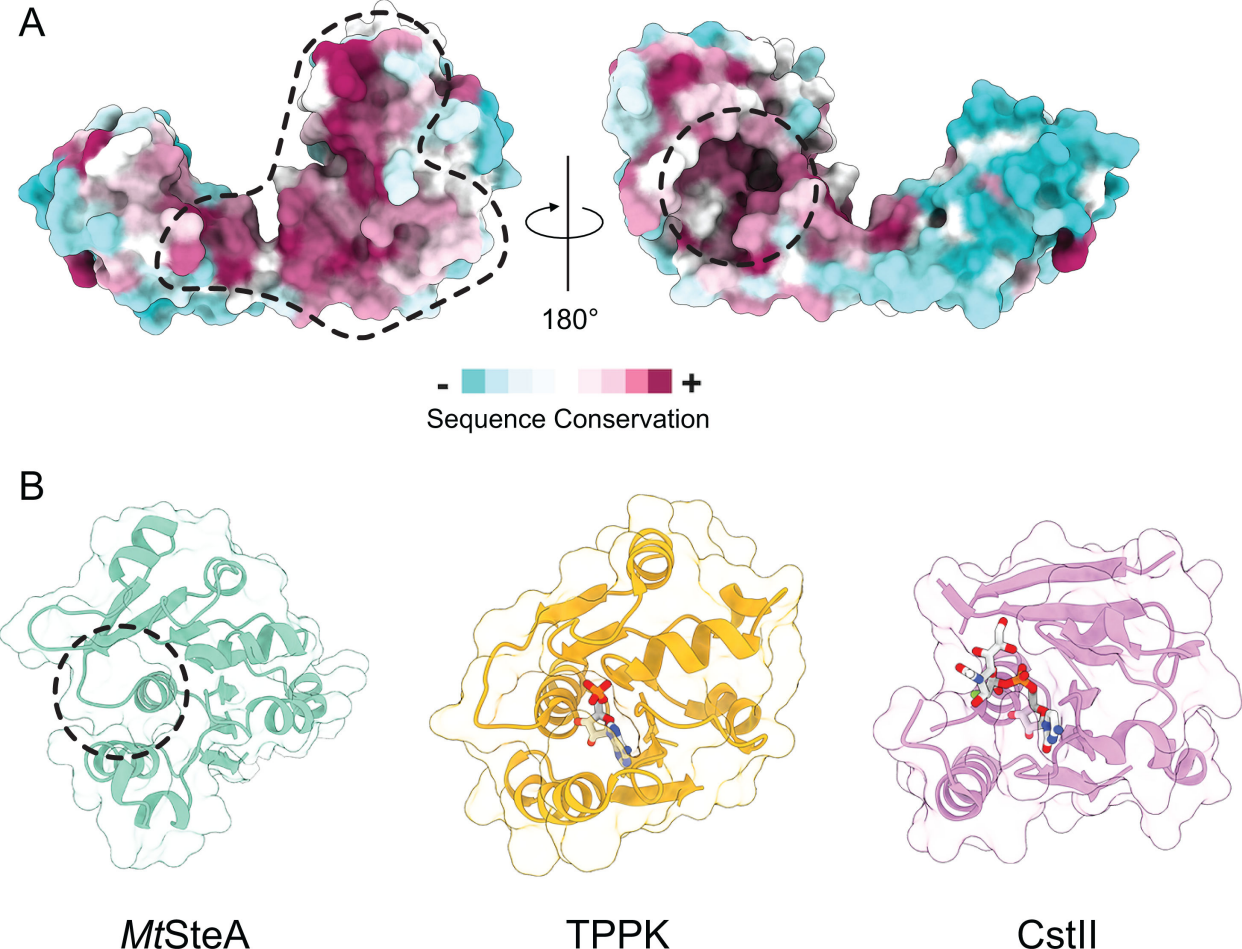
SteA is a putative phosphonucleotide-binding protein. (**A**) Mapping of conserved residues on the molecular surface of one SteA monomer, as calculated by Consurf (28). The large, conserved patch in the left panel corresponds to the dimerization interface, and the smaller conserved patch in the right panel corresponds to a putative ligand-binding site. (**B**) The putative *Mt*SteA binding pocket (left) matches the phosphonucleotide-binding sites of TPPK in complex with AMP (PDB 2F17, center) and CstII in complex with CMP (PDB 1Ro7, right). The bound ligands are shown in stick representation.

Sequence conservation analysis of SteA homologs reveals two clearly distinct patches of conserved residues on the molecular surface of the monomer (Fig. 3A). The larger patch on the left matches the dimerization interface, indicating a conserved homodimerization mode across species. The second patch, on the opposite side of the monomer, corresponds to a solvent-accessible protein cleft in the C-terminal domain. In both *Mt*SteA and *Cg*SteA, this putative ligand-binding site is predicted to bind carbohydrate using PeSTo-Carbs (25), a deep learning approach trained on protein-carbohydrate interfaces (Fig. S9A). Structural homology searches using DALI (24) revealed that the SteA C-terminal domain is similar to the ATP-binding domain of thiamine pyrophosphokinase (TPPK, [26]) (RMSD of 1.0 Å for 40 equivalent Cα positions) and, to a lesser extent, to the CMP-binding domain of sialyl-transferase CstII from *Campylobacter jejuni* (CstII, [27]) (RMSD of 1.0 Å for 33 equivalent Cα positions). The structural superpositions of *Mt*SteA with both TPPK and CstII showed that the conserved SteA pocket corresponds to the phosphonucleotide-binding sites for AMP and CMP, respectively (Fig. 3B), strongly supporting a functional role of the SteA-binding site. We analyzed the binding of various phosphonucleotides to the soluble construct of *Mt*SteA using nano differential scanning fluorimetry (nanoDSF), but the results led to weak, non-specific protein destabilization, which proved inconclusive (Fig. S9B). In contrast, the same experiment on the closely related *Cg*SteA homolog revealed that the addition of GDP or UDP, but not ADP or triphosphate nucleotides, significantly stabilized the protein (Fig. S9C), suggesting that SteA might function as a specific phosphonucleotide-binding protein.

### A SteAB complex involved in RipA activation

**Fig 4:**
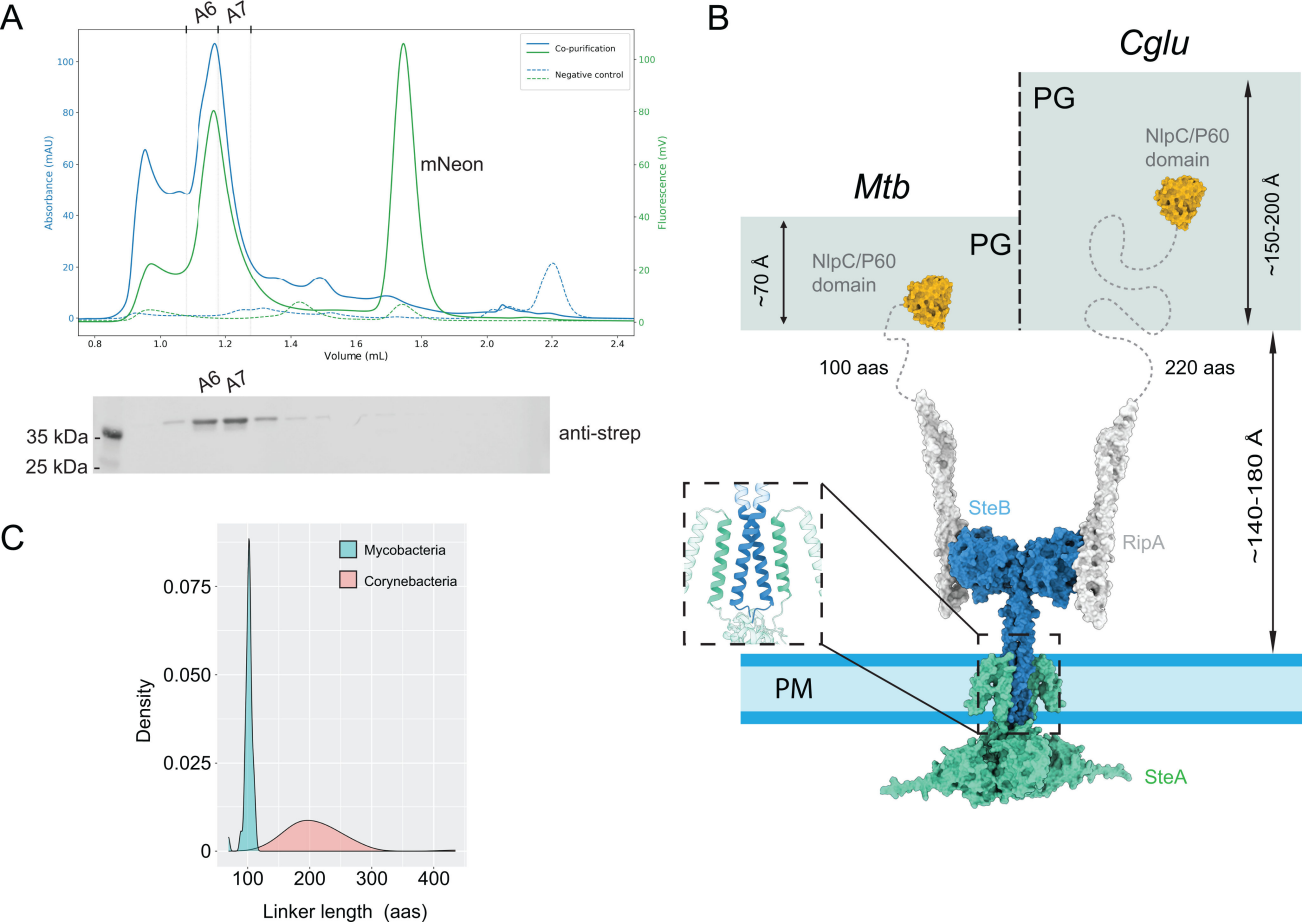
*Mt*SteAB-mediated regulation of *Mt*RipA. (**A**) Fluorescence-detection size-exclusion chromatography (FSEC) profile for the co-purified proteins mNeon-*Mt*SteA and *Mt*SteB-Strep (solid line) and the negative control (mNeon-SteA alone, dashed line). The blue trace indicates UV absorption, while the green trace indicates fluorescence. Fractions A6-A7 corresponding to a high-molecular weight symmetric peak, both in UV and fluorescence, are reported above the plot. Below the chromatogram, the anti-Strep western blot on the FSEC fractions confirms the presence of SteB-Strep in fractions A6-A7, indicating that mNeon-SteA and SteB-Strep co-elute in gel filtration. (**B**) Proposed model of the ternary complex of SteA, SteB, and RipA in an active conformation. The NlpC/P60 catalytic domain (orange) is connected to the N-terminal coiled-coil domain (white) by a flexible linker (dotted line). This connecting linker has different lengths in *Mtb* (shown at left) and *cglu* (shown at right), which correlates with the approximate thickness of the PG layer in these bacteria (30, 31). The inset shows the predicted TM 4-helix bundle. The inactive state (not shown) might be associated with a SteB conformation with a modified or disrupted CC, which would preclude the NlpC/P60 catalytic domain from reaching the PG substrate. (**C**) Distribution of connecting linker lengths in RipA homologs from *Mycobacteria* (cyan) and *Corynebacteria* (pink).

Previous research has shown that the two transmembrane proteins, SteA and SteB, form a complex that localizes to the cytokinetic ring in *Cglu* (13). In *Mtb*, the two homologous transmembrane proteins are encoded by adjacent genes, *Rv1697* (*steA*) and *Rv1698/mctB* (*steB*), and are expressed at similar levels in exponentially growing cells (220–250 protein copies per cell, [29]). To investigate whether *Mt*SteA and *Mt*SteB do interact with each other, we co-expressed full-length *Mt*SteA fused to the fluorescent mNeon protein (mNeon-*Mt*SteA) and full-length *Mt*SteB fused to the strep tag (*Mt*SteB-strep). The membrane fractions for the co-purified proteins and the negative control (absence of *Mt*SteB-strep) show a comparable fluorescence signal. However, in the elution fractions, the fluorescence signal is only retained when the two proteins are present, indicating that *Mt*SteA is retained on the resin due to its interaction with *Mt*SteB (Fig. 4A; Fig. S10A). The AF prediction of this complex revealed a (*Mt*SteA/*Mt*SteB)_2_ heterotetramer (Fig. S10B), in which the soluble cores of SteA and SteB on either side of the plasma membrane retain the same homodimeric arrangement as seen in their respective crystal structures. The SteA-SteB interaction is predicted to occur primarily through their TM regions, assembled into a four-helix bundle.

Integrating the *Mt*SteAB heterotetramer with the crystal structure of the *Mt*SteB*-Mt*RipA_CC_ complex described above provides a three-dimensional model of the ternary SteA/SteB/RipA system (Fig. 4B). According to this model, the coiled-coil regions of both SteB and the attached RipA are aligned nearly perpendicular to the membrane plane, positioning the NlpC/P60 catalytic domain deep within the periplasmic space, which possibly corresponds to the active enzyme state. This configuration enables the NlpC/P60 catalytic domain not only to physically interact with its PG substrate but also to deeply penetrate the porous PG layer, which has an average width of approximately 70 Å in *Mtb*, due to a relatively long linker (~100 residues) connecting the *Mt*SteB-bound RipA_CC_ to the C-terminal NlpC/P60 domain in *Mt*RipA (Fig. 4B). Interestingly, this linker length is well-conserved among mycobacterial RipA homologs, but it is consistently longer in corynebacterial homologs (Fig. 4C), matching a thicker PG layer in these species (~150–180 Å in *Cglu*, Fig. 4B).

### Conservation of the SteAB system in (and beyond) *Actinobacteria*

**Fig 5:**
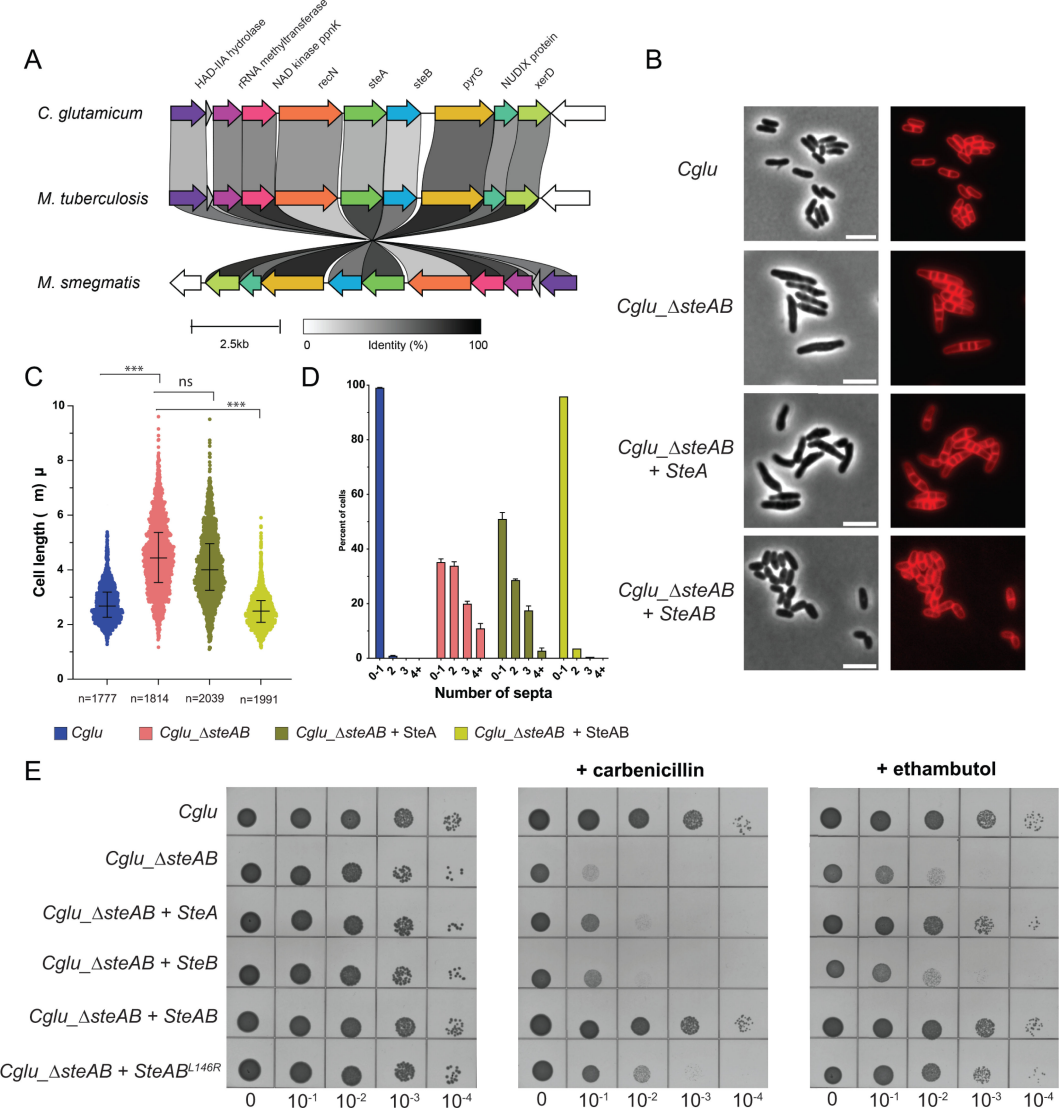
SteA/B depletion and complementation in *C. glutamicum*. (**A**) Clinker-generated (32) gene cluster comparison around *steA* and *steB* in *cglu*, *Mtb,* and *M. smegmatis*. (**B**) Representative images in Phase contrast (left) and membrane staining (Nile red, right) for the indicated strains. Scale bars = 5 μm. (**C**) Violin plots showing the distribution of cell length (Cohen’s *D*, from top to bottom: [***, = 1,65, *P* ~ 0], [ns, *d* = 0,29, *P* = 2,44e-19], [***, *d* = 1,96, *P* ~ 0]); the whiskers indicate the 25th to the 75th percentile, and the middle line the median. (**D**) Frequency histogram showing the number of septa per cell for the different strains, calculated from three independent experiments for each strain. Bars represent the mean ± SD. (**E**) Antibiotic sensitivity assays. BHI overnight cultures of the indicated strains were normalized to an OD600 of 0.5, serially diluted 10-fold, and spotted onto BHI agar medium with or without 1 µg/mL carbenicillin or 0.3 µg/mL ethambutol. In this panel, the asterisk in SteAB* indicates the point mutation Leu146-Arg in *Cg*SteB, which abolished formation of the RipA-SteB complex *in vitro* and is structurally equivalent to Leu147 in *Mt*SteB (see Fig. 1E, inset).

The occurrence of SteA and SteB is widespread and uneven across the *Actinobacteria* phylogeny (Fig. S11). Both proteins were identified in basal lineages, including *Rubrobacterales*, which is consistent with an ancestral origin, and they are also present in all analyzed *Mycobacteriales* genomes. Some of the lineages in which both SteA and SteB are absent correspond to very small, streamlined genomes, as in *Candidatus Actinomarinales*, *Candidatus Nanopelagicales*, and *Bifidobacterium*, which preferentially retain essential housekeeping functions. In most genomes where one gene is detected, the other is also present, with a few exceptions from incomplete genome assemblies (Fig. S11), where the apparent absences may reflect assembly artifacts or incompleteness rather than true gene loss. Co-occurrence of the two adjacent genes is even observed beyond *Actinobacteria*, in a restricted subset of *Bacillota* (mainly *Clostridia*), where they are found immediately downstream of the master regulator of sporulation, Spo0A, suggesting a possible role of SteAB in this process. Interestingly, in some taxa, especially within *Nocardioidaceae*, *steA* and *steB* occur as a single gene fusion (Fig. S12), strongly supporting a functional link between the two proteins. When present, *steA* and *steB* usually co-localize within the same genomic region. Notably, in *Mycobacteriales*, the local synteny is highly conserved, as illustrated in Fig. 5A by the comparison between *Mtb, Cglu,* and *M. smegmatis*.

### Coordinated and synergistic action of SteA and SteB in cell wall integrity

To understand the role of SteA and SteB *in vivo*, we used homologous recombination (33) to generate a *ΔsteAB* depletion strain in *C. glutamicum* ATCC13032 (*Cglu_ΔsteAB*), which we chose as a model organism for *Mycobacteriales*. As *steA* and *steB* are the last genes of the predicted 8-gene operon (34) that contains several putative transcription start sites, one of which overlaps with the *steA* coding sequence (Fig. S13A), we hypothesized that the single removal of *steA* would also silence *steB* through polar effects. The absence of *Cg*SteB was confirmed by anti-SteB western blot (Fig. S13B). The *Cglu*_Δ*steAB* strain resulted in elongated, multiseptal cells (Fig. 5B through D), as previously described for the mutants of the individual genes (13). We found that the ectopic expression of *Cg*SteA alone was not sufficient to restore the wild-type *Cglu* phenotype, and only the expression of both *Cg*SteA and *Cg*SteB fully complemented the mutant strain (Fig. 5B through D).

As endopeptidase defects have been associated with an increased sensitivity to cell-wall targeting β-lactam antibiotics in *Mycobacteriales* (3, 7), we tested the *Cglu*_Δ*steAB* mutant strain for carbenicillin sensitivity. The mutant displayed a high antibiotic sensitivity, phenocopying the increased susceptibility to carbenicillin of RipA-depleted *M. smegmatis* strains (3) and establishing another functional link between SteAB and RipA. Wild-type-like carbenicillin resistance could only be restored upon ectopic expression of both *Cg*SteA and *Cg*SteB, but not when expressing the individual proteins (Fig. 5E). To investigate if this phenotype could be due to the SteA/B-mediated control of RipA, we produced the Leu146-Arg point mutant of *Cg*SteB (*Cglu_*SteB_L146R_), which was previously shown to abolish the SteB-RipA interaction *in vitro* (12). We observed that the ectopic expression of *Cg*SteA/*Cg*SteB, but not that of *Cg*SteA/*Cg*SteB_L146R_, restored wild-type-like carbenicillin tolerance in the *Cglu*_Δ*steAB* strain, even if SteB_L146R_ ectopic expression levels were comparable to those of wild-type *Cg*SteB (Fig. S13B). As *steA* and *steB* were first identified in a transposon mutagenesis screening to identify genes associated with sensitivity to ethambutol (13), we also observed a hypersusceptibility to ethambutol in the *Cglu_ΔsteAB* strain (Fig. 5E). Surprisingly, however, the ectopic expression of *Cg*SteA alone was sufficient to restore nearly wild-type-like ethambutol tolerance (Fig. 5E), suggesting that SteA may have an additional SteB-independent role, possibly mediated by protein-protein interactions with other septal components of the divisome, such as for instance the recently proposed interaction in *Mtb* with the MtrAB two-component system involved in cell-wall homeostasis (35). Our findings in *Cglu* might explain why *Mt*SteA, but not *Mt*SteB, is essential for growth in *Mtb* (16–19).

## DISCUSSION

The structural characterization of the SteAB complex and its interactions with RipA provides important functional insights into the mechanism by which this system controls daughter cell separation in *Mtb*. The cytoplasmic domain of *Cg*SteA can bind GDP- or UDP-containing molecules, pointing to a possible source of power to trigger SteAB-mediated RipA activation. Although further work is required to identify the specific ligand, different intermediate metabolites and recycling molecules from cell wall synthesis do contain this class of phosphonucleotide moieties (36, 37). This suggests that the system may be able to sense the status of the cell wall to coordinate cytokinesis. The cytoplasmic activation signal might then be transmitted through the SteAB TM helical bundle to modulate periplasmic RipA recruitment and activation by directly affecting the productive positioning of the catalytic domain for PG hydrolysis. Both four-helix bundles and two-helical CCs are ubiquitous sensory modules involved in bacterial signal transduction (38). Indeed, the proposed SteAB mechanism is reminiscent of those described for bacterial TM histidine kinases, where a conformational signal transmitted along the membrane-connecting two-helical coiled-coil serves as a switching mechanism to control enzyme activity (39–41). As both SteA and SteB have been reported to interact with other divisome proteins (42, 43), we cannot exclude the possibility that additional septal proteins—yet to be identified—could transiently interact with the TM SteAB helical bundle or the cytoplasmic domain of SteA to fine-tune signal transduction.

It was previously proposed that *Mt*SteB (MctB) could be an outer membrane porine involved in copper transport (15). Shortly thereafter, however, the same researchers suggested that the protein might be anchored to the inner membrane and could fulfill a more pleiotropic role (44, 45). Our findings confirm and expand on the second hypothesis. A primary role of *Mt*SteB in the control of daughter cell separation can explain the severe growth defects observed for a Δ*steB Mycobacterium smegmatis* mutant strain or the reduced virulence of a SteB-deficient *Mtb* mutant strain in mice and guinea pigs, originally attributed to copper toxicity (15). Several lines of evidence support the involvement of the SteAB system in cell division: both SteA and SteB were identified as direct or indirect interaction partners of two core divisome proteins, FtsB and FtsQ, in *Mycobacteria* (42, 46); the disruption of a cell division membrane protein in *Mtb* resulted in the increased transcription of both *steA* and *steB* (47); the inhibition of SteA expression in *Mtb* produced long chains of cells with unresolved septa, suggesting an impairment in the final stages of cell separation (35); a SteA-deficient *Mycobacterium abscessus* strain also displayed a multi-septa phenotype and higher antibiotic susceptibility (48); and transposon insertion mutants of *steA* in *Mycobacterium avium* led to cell wall modifications and reduced multidrug resistance (49).

**Fig 6:**
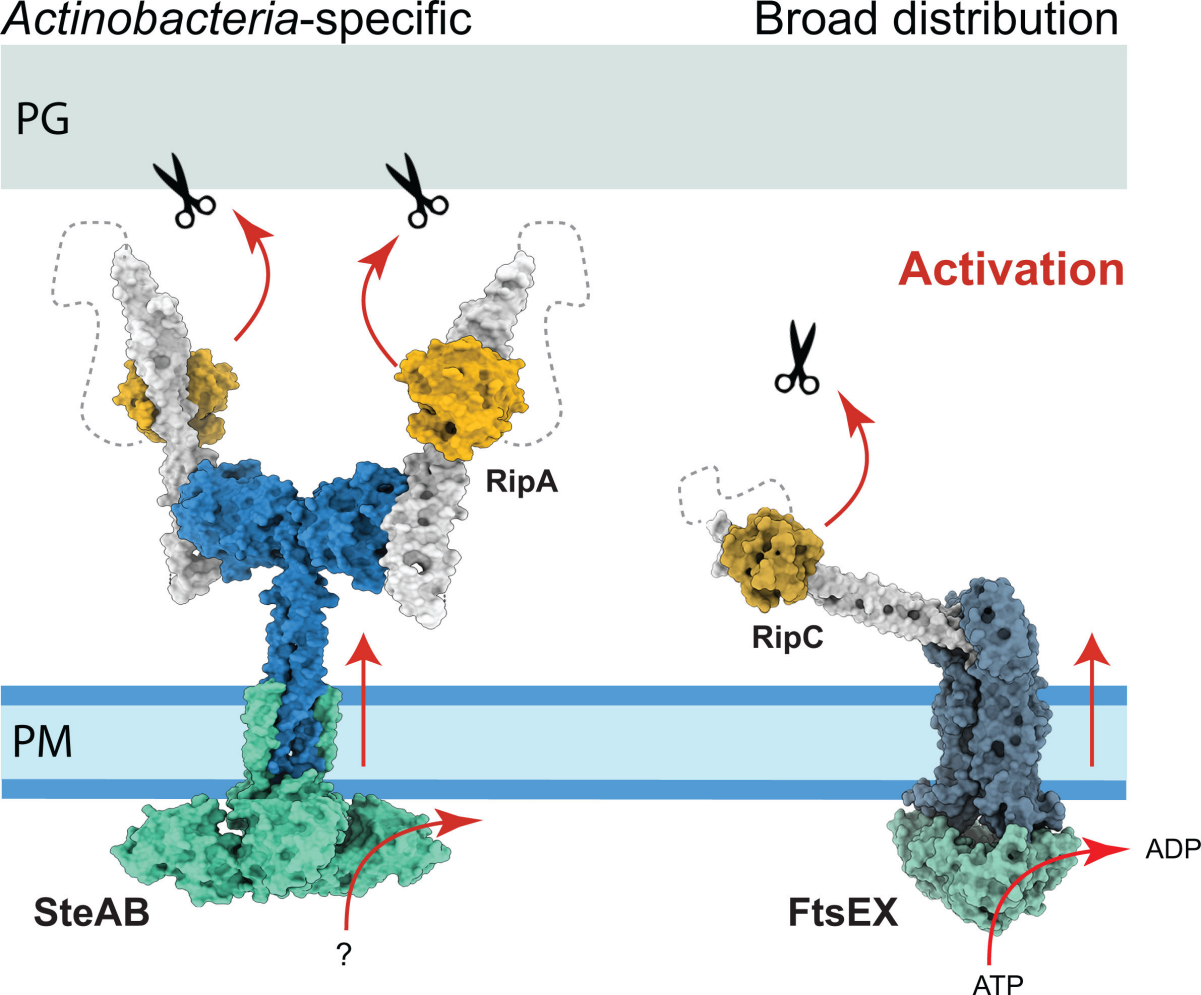
Two regulatory systems of PG hydrolysis in *Mtb*. Overall views of the SteAB-RipA complex (left panel) and the FtsEX-RipC complex (right panel, PDB 8JIA, [53]). The NlpC/P60 catalytic domains (shown in orange) are shown in their autoinhibited state, with their active sites bound to their respective N-terminal coiled-coil domains (in white). In both cases, phosphonucleotide binding to the cytoplasmic domain (SteA or FtsE) would draw the respective NlpC/P60 domains toward the PG layer, where the physical interaction with substrate and/or a conformational signal propagated across the membrane would release the catalytic domain for PG hydrolysis (as illustrated in Fig. 4A for RipA).

In addition to the SteAB system, the ABC transporter-like FtsEX is also involved in the regulation of PG hydrolysis in *Mtb* (50). FtsEX controls the action of RipC, another NlpC/P60 hydrolase with a domain organization similar to RipA but whose physiological role remains unclear (51, 52). The recent cryo-EM structure of the FtsEX-RipC complex (53) revealed that, unlike RipA, whose CC domain is roughly perpendicular to the membrane, RipC binds at a largely inclined angle with respect to the central axis of FtsEX (Fig. 6). A common feature of these two systems is that the two NlpC/P60 catalytic domains are autoinhibited by their own N-terminal CC domains. In both cases, enzyme activation relies on the productive periplasmic positioning of the NlpC/P60 domain, as the activation of RipC by FtsEX causes its CC domain to tilt toward the PG layer (53), favoring the physical interaction of the catalytic domain with its substrate. However, these two systems differ markedly in their overall architectures, mechanisms of action, and biological roles. FtsEX belongs to subfamily VII of ABC transporters and uses ATP hydrolysis as the power source to control PG hydrolysis. In contrast, SteAB presents a novel architecture, with a cytoplasmic moiety partially resembling TPPKs that might bind phosphonucleotide-containing molecules but not ATP, and a periplasmic moiety that constitutively recruits RipA.

FtsEX is highly conserved in bacteria, suggesting an ancient general role in PG remodeling. In contrast, SteAB is largely restricted to *Actinobacteria* and especially conserved in *Mycobacteriales*. This group of bacteria has a thick waxy cell envelope formed by the plasma membrane, a two-layered cell wall composed of PG and arabinogalactan, and an unusual outer membrane composed of mycolic acids, the mycomembrane. As the cell envelope is sequentially assembled at the septal junction, the peripheral PG layer remains continuous, acting as a mechanical link that holds the daughter cells together throughout septation (2). The primary role of RipA is to cleave this stress-bearing PG layer, asymmetrically weakening the mechanical strength of the cell envelope (4, 5) to generate a breaking point for V-snapping. This rapid, mechanically driven cell separation promoted by turgor pressure (2, 54) is a common trait in *Mycobacteriales*, but rare in other bacteria (55). Accordingly, PG hydrolase deficiency results in linear chains of non-growing cells that can be induced to divide by the local application of external mechanical forces (56). Understanding these specific mechanisms of cell wall remodeling and cytokinesis could offer new attractive therapeutic targets in the context of a critical human pathogen, *M. tuberculosis*.

## MATERIALS AND METHODS

### Bacterial strains and growth conditions

*Escherichia coli* DH5α or CopyCutter EPI400 were used for cloning and grown in Luria-Bertani (LB) broth or agar plates at 37°C supplemented with 50 µg/mL kanamycin or 100 µg/mL carbenicillin when required. For protein production, *E. coli* BL21 (DE3) (for soluble proteins) and C41 (57) (for membrane proteins) cells were grown in 2YT broth supplemented with 50 µg/mL kanamycin or 100 µg/mL carbenicillin at the appropriate temperature. For the *in vivo* experiments, *Cglu ATCC13032* was defined as the wild-type (wt) strain. All *Cglu* strains generated for this study (Table S2) were grown at 30°C with shaking at 120 rpm in brain heart infusion (BHI) medium or CGXII minimal medium supplemented with 4% sucrose (58). When required, the BHI and CGXII media were supplemented with 25 µg/mL kanamycin (BHI_kan_ and CGXII_kan_).

### Knock-out strain generation of *C. glutamicum*

*CgluΔsteAB* was generated using a two-step recombination strategy with the *pk19mobsacB* plasmid to delete the *steA* coding region as described previously (33). Briefly, approximately 600 bp flanking *steA* upstream and downstream of *Cglu* genomic DNA were amplified by PCR using chromosomal DNA of *Cglu* as a template. The PCR fragments were cloned by Gibson assembly into a linearized *pk19mobsacB*. The resulting plasmid was electroporated into *Cglu*. Successful first recombination events were confirmed by PCR, and positive colonies were grown overnight in BHI_kan_ medium. The second round of recombination was selected by growth in BHI plates containing 10% (wt/vol) sucrose. Kanamycin-sensitive colonies were screened by colony PCR to check for *steA* deletion. Positive colonies were verified by sequencing (Eurofins, France).

### Cloning for recombinant protein production

The primers used for PCR amplification of the different fragments or site-directed mutagenesis are listed in Table S3. Cloning was performed by assembling the purified PCR fragments into the specified derivative expression vectors using the commercially available NEBuilder HiFi DNA Assembly Cloning Kit (New England Biolabs). For soluble protein production, *M. tuberculosis steA, steB,* and *ripA* truncations were amplified by PCR using codon-optimized synthetic genes (GenScript) and cloned into a pET vector containing an N-terminal 6xHis-SUMO tag. For full-length protein production, the *Mtb steA* and *steB* genes were amplified by PCR as above and cloned into the *pTGR5* shuttle expression vector (59) under control of the *PgntK* promoter. *Mt*SteA was cloned in frame with an N-terminal Alfa-mNeonGreen tag, and *Mt*SteB was cloned with a C-terminal Strep tag. A co-expression vector containing Alfa-mNeonGreen-*Mt*SteA and *Mt*SteB-Strep, each under the control of its own *PgntK* promoter, was generated by restriction-ligation cloning.

### Soluble protein expression and purification

All constructs were expressed in *E. coli* BL21 (DE3) using an autoinduction method (60). After an initial incubation of 4 h at 37°C, the cells were grown for 20 h at 18°C in 2YT medium complemented with autoinduction supplement and 100 μg/mL carbenicillin. Cells were harvested, flash frozen in liquid nitrogen, and stored at −20°C. Cell pellets were resuspended in lysis buffer (50 mM Hepes pH 8.0, 500 mM NaCl, 10 mM imidazole, 5% glycerol, 1 mM MgCl_2_, benzonase, lysozyme, 0.25 mM Tris (2-carboxyethyl) phosphine hydrochloride (TCEP), EDTA-free protease inhibitor cocktails [Roche]) at 4°C and lysed by sonication. Cell debris was removed by centrifugation (15,000 × *g*) for 15 min at 4°C, and the supernatant loaded onto a Ni-NTA affinity chromatography column (HisTrap FF crude, Cytiva) pre-equilibrated in buffer A (50 mM Hepes pH 8, 500 mM NaCl, 10 mM imidazole, and 5% glycerol). His-tagged proteins were eluted with a linear gradient of buffer B (50 mM Hepes pH 8.0, 500 mM NaCl, and 0.5 M imidazole). The fractions of interest were pooled and dialyzed in the presence of the SUMO protease at a 1:100 wt/wt ratio. Dialysis was carried out at 4°C overnight in SEC buffer (25 mM Hepes pH 8.0 and 150 mM NaCl). For *Mt*SteA, a higher salt concentration (500 mM NaCl) is necessary to keep the protein soluble. Cleaved His-tags and His-tagged SUMO protease were removed with Ni-NTA agarose resin. The cleaved protein was concentrated and injected onto a Superdex 75 or 200 16/60 size exclusion column (GE Healthcare) pre-equilibrated at 4°C in SEC buffer. The peak corresponding to the protein was concentrated, flash frozen in small aliquots in liquid nitrogen, and stored at −80°C. Protein concentration was determined spectrophotometrically at 280 nm, and purity was confirmed by sodium dodecyl sulfate–polyacrylamide gel electrophoresis (SDS-PAGE).

Se-Met-derived *Cg*SteA was expressed in *E. coli* BL21 (DE3) with all media containing 50 µg/mL carbenicillin. Cells were grown for 8 h at 37°C in 2YT medium and inoculated 1:100 in M9 medium (33.7 mM Na_2_HPO_4_-2H_2_O, 22.0 mM KH_2_PO_4_, 8.6 mM NaCl, 9.4 mM NH_4_Cl, 2 mM MgSO_4_, 0.3 mM CaCl_2_ 0.4% (wt/vol) D-glucose, 3.8 µM thiamin, and 4.1 µM biotin). The overnight culture was diluted 1:50 in fresh M9 medium and grown until OD600 = 0.6. The methionine biosynthetic pathway was inhibited by adding lysine, phenylalanine, and threonine at 100 mg/L; isoleucine and valine at 50 mg/L; and selenomethionine at 60 mg/L. Protein expression was induced 30 min after addition of amino acids by adding IPTG to a final concentration of 1 mM, and the cells were grown for 20 h at 18°C, harvested, and flash frozen in liquid nitrogen. Protein purification was performed as described above.

### Membrane protein expression and purification

mNeon-*Mt*SteA and *Mt*SteB-Strep full-length proteins were recombinantly co-expressed in *E. coli* BL21 strain. Cells were grown overnight in 3 L of 2YT media at 30°C, and proteins were expressed using the *PgntK* promoter. Cells were harvested and flash-frozen in liquid nitrogen. All following steps were performed at 4°C unless otherwise specified. Cell pellets were resuspended in Lysis buffer (50 mM Hepes pH 8, 500 mM NaCl, 10% glycerol, 1 mM MgCl_2_, benzonase, lysozyme, and EDTA-free protease inhibitor cocktails [Roche]) and lysed through 2× passages in a CellD press (Constant Systems) at 2.2 kbar. The lysate was cleared by centrifugation (15 min, 15,000 × *g*) and centrifuged in Ti45 tubes for 1 h at 100,000 × *g* in an Optima L-100 XP ultracentrifuge (Beckman Coulter). Pelleted membranes were resuspended with a Dounce homogenizer in 4 mL of Membrane buffer (50 mM Hepes pH 8, 500 mM NaCl, 10% glycerol, and 1.2% [wt/vol] DDM) and solubilized under gentle rotation for 30 min at room temperature; 100 µL of Strep-Tactin XT 4 Flow high-capacity resin equilibrated in Membrane buffer were added to the solubilized membrane fraction. After 30 min of incubation with gentle rotation at 4°C, the sample was applied onto a Pierce spin column, and the flow-through was discarded by centrifugation at 300 × *g*. The resin was washed 5 times with 500 µL of Wash buffer (50 mM Hepes pH 8, 500 mM NaCl, 10% glycerol, and 0.02% DDM) and then eluted 4 times in 50 µL Elution buffer (25 mM Hepes pH 8, 150 mM NaCl, 10% glycerol, 50 mM biotin, and 0.02% DDM). The elution fractions were analyzed by fluorescence detection (Alexa 488 filter) using a ChemiDoc MP Imaging System and then pooled and concentrated to 50 µL. The concentrated elution fractions were analyzed by fluorescence-detection size-exclusion chromatography (F-SEC) using an ÄKTA Micro liquid chromatography system (Cytiva) equipped with a FR-20Axs fluorescence detector (Shimadzu). Samples were loaded onto a Superdex 200 Increase 3.2/300 size-exclusion column (Cytiva) equilibrated in SEC buffer (25 mM Hepes pH 8, 150 mM NaCl, and 0.02% DDM) and eluted at flow rate of 0.04 mL/min at 4°C. In-line 280 nm absorbance and fluorescence (Excitation 490 nm, Emission 525 nm) were monitored to assess total protein elution and detect mNeon fluorescence, respectively. The same expression/purification protocol was executed in parallel for a BL21 strain expressing only mNeon-*Mt*SteA as a negative control.

### Analytical SEC

The oligomerization state of *Cg*SteB_ΔTM_ was probed using a Superdex-200 3.2/300 column equilibrated in buffer 25 mM Hepes pH 8, 150 mM NaCl. 50 µL of pure protein sample was injected on the column, and the elution peak was checked by SDS-PAGE. The calibration curve is based on the BioRad gel filtration standard containing: Thyroglobulin (670 kDa), γ-globulin (158 kDa), Ovalbumin (44 kDa), Myoglobin (17 kDa), and Vitamin B12 (1.350 kDa).

### SEC-SLS

The oligomerization state of SteA was determined by SEC coupled to a triple detection (concentration detector: UV detector, refractometer; SLS 7°, 90°; viscometer) on a Omnisec RESOLVE and REVEAL instrument (Malvern Panalytical). SteA (100 µL sample at 1–5 mg/mL) was centrifuged for 15 min at 27,000 × *g* and injected on a Superdex 75 Increase 10/300 GL column (GE) pre-equilibrated in 25 mM Hepes pH 7.5, 150 mM NaCl at 20°C. External calibration was done by injecting 10 µL bovine serum albumin (BSA) at 18.3 mg/mL. The refractive index, static light scattering, and viscosity measurements were processed to determine the mass average molecular mass and the intrinsic viscosity using the OMNISEC V11.32 software (Malvern Panalytical, UK).

### Protein crystallization and data collection

Screening for initial crystallization conditions was carried out by the sitting drop vapor diffusion method using a MosquitoTM nanoliter-dispensing system (*TTP Labtech, Melbourn, United Kingdom*), and the established protocols adhered to the Crystallography Core Facility of the Institut Pasteur (61). Promising hits were then reproduced and optimized manually using the hanging drop vapor diffusion method. All crystallization experiments were carried out at 18°C. *Mt*SteA (5 mg/mL) crystals grew directly in the purification buffer, 0.5 M NaCl, 25 mM Hepes pH 8, without any further manipulation. *Mt*SteB (11.4 mg/mL) crystals were obtained in 0.1 M CdCl_2_, 0.1 M Na Acetate pH 4.6, and 30% PEG 400. Crystals of the complex between *Mt*SteB-*Mt*RipA_CC_ were obtained in 0.01 M CoCl_2_, 0.1 M Na Acetate pH 4.6, and 1 M Hexane-1,6-diol. An equimolar solution (200 μM, final concentration) of the two proteins was incubated on ice for 30 min prior to the crystallization experiment. Crystals of CgSteA (10 mg/mL) were grown in 0.1 M imidazole 8 pH, 0.2 M calcium acetate, 10% wt/vol PEG 8K, and those of SeMet-derived *Cg*SteA (10 mg/mL) were obtained in 0.1 M Tris pH 8, 0.2 M CaCl_2_, 0.6 M LiCl, and 18% PEG 3350. Upon briefly soaking in a cryo-protectant solution containing the mother liquor supplemented by 33% (vol/vol) glycerol or PEG 400, crystals were flash frozen in liquid nitrogen. Diffraction data were collected at 100K at the Synchrotron facilities Soleil (Saclay, France) or ESRF (Grenoble, France).

### Structure determination and crystallographic refinement

All diffraction data were processed using XDS (62) and Aimless from the CCP4 software suite (63) using the AutoPROC workflow (64). Crystals of the *Mt*SteB-RipA_CC_ complex showed high solvent content (73%) and a strong anisotropy. Anisotropy corrections with STARANISO (65) were applied to the diffraction data from *Cg*SteA, *Mt*SteA, and *Mt*SteB-RipA_CC_ crystals, resulting in a lower data completeness at high resolution (Table S1).

Structure determination of *Mt*SteB was carried out using cadmium SAD phasing (cadmium was present in the crystallization solution) on a monoclinic crystal form at 2.5 Å resolution. The CRANK2 pipeline (66) within the CCP4 software suite was used to identify 24 cadmium sites and produce a model of the protein with six molecules in the asymmetric unit. This model was used as a search probe to solve the 2 Å resolution orthorhombic *Mt*SteB crystal form, with three independent protein molecules in the asymmetric unit. The structure of the *Mt*SteB-RipA_CC_ complex was solved by molecular replacement using *Mt*SteB as search model. Despite strong anisotropy and high solvent content (>70%), the initial Fourier maps provided enough information to unambiguously build the missing *Mt*RipA_CC_ molecule. The *Mt*SteA structure was solved by molecular replacement using the AlphaFold predicted coordinates of the N and C-terminal domains, separately. Twenty residues corresponding to the central alpha-helix linker were removed and manually re-built in Coot to account for the relative flexibility of the two domains. The structure of *Cg*SteA was determined by SAD phasing at 3 Å resolution using the SeMet-labeled protein and refined against a 2 Å data set from native *Cg*SteA crystals.

All crystal structures underwent extensive iterative cycles of manual model building with COOT (67) and reciprocal space refinement with PHENIX (68) or BUSTER (69), leaving aside 5% of the observed reflections for Rfree calculation. Non-crystallographic symmetry (when appropriate) and translation-libration-screw (TLS) constraints were applied during refinement. The final refinement statistics are reported in Table S1. All molecular graphics images were generated using ChimeraX (70).

### AlphaFold predictions

Structural predictions were performed on the High-Performance Computing (HPC) Core Facility of the Institut Pasteur computer cluster using a local installation of AlphaFold2 (v2.3.2). Model type “monomer” or “multimer” was used for monomeric and multimeric predictions, respectively. To ensure unbiased predictions for the different RipA forms, structural templates were disabled in these cases. All other parameters were kept as default and amber relaxation was applied to all output models. All models converged to similar conformations, and only the best one for each run is shown in Fig. S2, S5, and S9.

### BLI assays

The affinities of purified *Mt*SteB and *Mt*RipA catalytic domain (*Mt*RipA_CAT_, residues 261–472) toward the *Mt*RipA coiled coil domain (*Mt*RipA_CC_, residues 40–240) were assessed in real-time using a bio-layer interferometry Octet-Red384 device (Pall ForteBio) at 25°C. Biotinylated *Mt*RipA_CC_ was diluted at 10 μg/mL in buffer A (25 mM HEPES pH 7.5, 150 mM NaCl, and 5% glycerol) and immobilized on the commercially available Sartorius Streptavidin biosensors for 5 min at 1,000 rpm, followed by a washing step in buffer A for 3 min to remove any loosely bound protein. For the biotinylation reaction, 100 μL of recombinant *Mt*RipA_CC_ (25 μM) was incubated with 20 molar excess of EZ-Link NHS-PEG4-Biotin (Thermo Scientific) following supplier instructions.

Empty sensors were used as reference for unspecific binding. *Mt*RipA_CC_-loaded or empty reference sensors were incubated for 5 min at 1,000 rpm in the absence and presence of serially diluted concentrations of *Mt*SteB (200–3.125 μM range) or *Mt*RipA_CAT_ (80–1.25 μM range) in buffer A supplemented with BSA at 1 mg/mL and 0.05% Tween 20. Specific signals were obtained by double referencing, subtracting both non-specific signals measured on empty sensors and buffer signals on biotinylated *Mt*RipA_CC_-loaded sensors. Two or three independent experiments were performed for *Mt*SteB and *Mt*RipA_CAT_, respectively, and *Kd* values were obtained from steady-state signal versus concentration curves fitted with GraphPad Prism 9 assuming a one-site binding model.

### Nanoscale differential scanning fluorimetry (NanoDSF) assay

Fluorescence measurements were carried out on the Prometheus NT.48 (NanoTemper Technologies) using standard-grade glass capillaries filled with 10–12 μl of *Mt*SteA or *Cg*SteA at 40 μM in 25 mM HEPES, pH 8, 500 mM NaCl, and 5% glycerol. Prior to the experiment, protein samples were centrifuged at 15,000 × *g* for 15 min to remove any large aggregate. Nucleotide stock solutions were prepared in water adjusting the pH to ~7.5 with diluted NaOH and added at a final concentration of 8 mM. An excitation power of 100% and a temperature ramp from 15°C to 95°C with a slope of 1°C/min were used. The Tm was determined in the PR.ThermControl software as the maximum of the first derivative for the ratiometric (F350/F330) melting curves. All melting experiments were performed at least in triplicates.

### Antibiotic susceptibility assay

Overnight cultures of *Cglu* strains were diluted to OD_600_ = 0.5 in fresh BHI_kan_ medium and serially diluted 1:10 in the same medium; 5 µL of each dilution was spotted on BHI_kan_ plates with or without 1 µg/mL carbenicillin or 0.3 µg/mL ethambutol. Plates were imaged on a ChemiDoc Imaging System (Bio-Rad) after 28 h of growth at 30°C.

### Phase contrast and fluorescence microscopy

For imaging, cultures were grown in BHI at 30°C for around 6 h, pelleted at 5,200 × *g* at RT and inoculated into CGXII, 4% sucrose, and kanamycin (25 μg/mL) for overnight growth. The following day, cultures were diluted to OD_600_ 1 in CGXII, 4% sucrose (+/− 1% gluconate) and grown for about 7 h at 30°C to an OD_600_ of about 5 (early exponential phase). For each sample, 100 μL of culture were pelleted and washed twice with fresh medium. For membrane staining, Nile Red (Enzo Life Sciences) was added to the culture (2 μg/mL final concentration) just prior to placing them on 2% agarose pads prepared with the corresponding growth medium. Cells were visualized using a Zeiss Axio Observer Z1 microscope fitted with an Orca Flash 4 V2 sCMOS camera (Hamamatsu) and a Pln-Apo 63X/1.4 oil Ph3 objective. Images were collected using Zen Blue 2.6 (Zeiss) and analyzed using the Fiji software (71), custom trained Omnipose (72) and MicrobeJ (73).

### Statistics and reproducibility

Because of the important number of cells analyzed in each sample, Cohen’s *d* value was used to describe effect sizes between different strains independently of sample size:


$$\;d=\;\frac{mean_{2}-\;mean_{1}}{\sqrt{\frac{\left(n_{1}-1\right)*SD_{1}^{2}+\left(n_{2}-1\right)*SD_{2}^{2}}{n_{1}+n_{2}-2}}}$$

Values were interpreted as previously described (74), briefly the intervals of reference are considered: small (ns), *d* < 0.50; medium (*), 0.50 < *d* < 0.80; large (**), 0.80 < *d* < 1.20; very large (***), 1.20 < *d* < 2.0; huge (****), *d* > 2.0.

Unless otherwise stated, *P* values were obtained by a Welch two-sample *t*-test calculated on R. All experiments were performed as biological triplicates. Some autofluorescence is observed for wild-*type Cglu* as previously described (75). All micrographs and blots shown are representative of similar experiments carried out at least three times.

### Antibody production and characterization and western blots

Polyclonal anti-SteA and anti-SteB antibodies were raised in rabbits (Covalab) against the purified soluble domains of *Cg*SteA and *Cg*SteB, respectively. For antibody purification, sera from day 67 post-inoculation were purified using a 1 mL HiTrap NHS-Activated HP column (GE Healthcare) loaded with the corresponding antigen according to the manufacturer’s instructions. Sera were diluted in binding buffer (20 mM Sodium Phosphate pH 7.4 and 500 mM NaCl), loaded onto the column, and washed with 7 mL of binding buffer. Antibodies were eluted with 10 mL elution buffer (100 mM Glycine pH 3 and 500 mM NaCl) and neutralized with 1M Tris pH 9. Purified antibodies were concentrated to 8 mg/mL and mixed 1:1 with glycerol 100%, aliquoted, and stored at −20°C. The characterization of the antibodies is shown in Fig. S14.

For western blots, bacterial pellets of cell extracts were resuspended in lysis buffer (50 mM Bis-Tris pH 7.4; 75 mM 6-Aminocaproic Acid; 1 mM MgSO4; Benzonase and protease Inhibitor) and disrupted at 4°C with 0.1 mm glass beads and using a PRECELLYS 24 homogenizer. Total extracts (from 60 μg to 120 μg) were run on an SDS-PAGE gel, transferred onto a 0,2 μm nitrocellulose membrane, and incubated for 1h with blocking buffer (5% skimmed milk, 1× TBS-Tween buffer) at room temperature (RT). Blocked membranes were incubated for 1 h at RT with the corresponding primary antibody diluted to the appropriate concentration in blocking buffer. After washing in TBS-Tween buffer, membranes were probed with an anti-rabbit or an anti-mouse horseradish peroxidase-linked secondary antibody (GE Healthcare) for 45 min. For chemiluminescence detection, membranes were washed with 1× TBS-T and revealed with HRP substrate (Immobilon Forte, Millipore). Images were acquired using the ChemiDoc MP Imaging System (Biorad). Dilutions used were anti-SteA (1:500), anti-SteB (1:1,000), and anti-rabbit secondary Abs (1:10,000).

### Synteny analyses

We obtained a proteomes database containing five representative species of all Actinobacteria from Petit et al. (74). We used HMM profile searches to identify proteins SteA and SteB in the protein database. First, we used the HMMER package (v3.3.2) (76) tool jackhmmer to look for homologs of *C. glutamicum* SteA and SteB in all the proteomes using the GenBank (77) sequences BAB98808.1 and BAB98809.1 as queries, respectively. The hits were aligned with linsi, the accurate option of mafft (v7.475) (78) and default parameters. The alignments were manually curated, removing sequences that did not align globally. The hits obtained by jackhmmer might not include sequences that are very divergent from the single sequence queries. For this reason, the alignments were used to create HMM profiles using the HMMER package (v3.3.2) tool hmmbuild. These specific and curated HMM profiles of SteA and SteB were used for the second and final rounds of searches against the proteomes using the HMMER tool hmmsearch. The new hits were aligned with linsi, the accurate option of mafft (v7.475). The results were mapped on a species phylogeny of Actinobacteria (71) using the online tool iTOL (79).

## Data Availability

Atomic coordinates and structure factors have been deposited in the PDB with accession codes 9HLE (*Mt*SteB), 9HMX (*Mt*SteB-RipA_CC_), 9HMY (*Mt*SteA), 9HMZ (*CgSteA*). All materials of this paper can be provided upon reasonable request.

## SUPPLEMENTAL MATERIAL

Supplemental material mbio.03700-25-s0001.pdf Figures S1 to S14, Tables S1 to S3, and legend for Video S1.

Video S1 mbio.03700-25-s0002.mp4 Flexibility of the SteA N-terminal domain.

ASM does not own the copyrights to Supplemental Material that may be linked to, or accessed through, an article. The authors have granted ASM a non-exclusive, world-wide license to publish the Supplemental Material files. Please contact the corresponding author directly for reuse.
