# Supplementary material for: Mechanistic insights into SteAB regulation of cell wall hydrolase RipA in Mycobacterium tuberculosis
Source: mBio. 2026 Jan 26;17(3):e03700-25. doi: 10.1128/mbio.03700-25 (PMC12977500; doi:10.1128/mbio.03700-25)
Supplement: Supplemental material — Figures S1 to S14, Tables S1 to S3, and legend for Video S1. [file mbio.03700-25-s0001.pdf]

Supplementary information for:

Mechanistic insights into SteAB regulation of the cell wall hydrolase RipA in *Mycobacterium tuberculosis*

Giacomo Carloni, Quentin Gaday, Daniela Megrian, Julienne Petit, Mariano Martinez, Adrià Sogues, Mathilde Ben Assaya, Marcell Kakonyi, Ahmed Haouz, Pedro M Alzari and Anne Marie Wehenkel

Correspondence to A.M. Wehenkel and P.M. Alzari

Email: anne-marie.wehenkel@pasteur.fr and pedro.alzari@pasteur.fr

This PDF file includes:

Figures S1 to S14

Tables S1 to S3

Legend to video V1

SI References

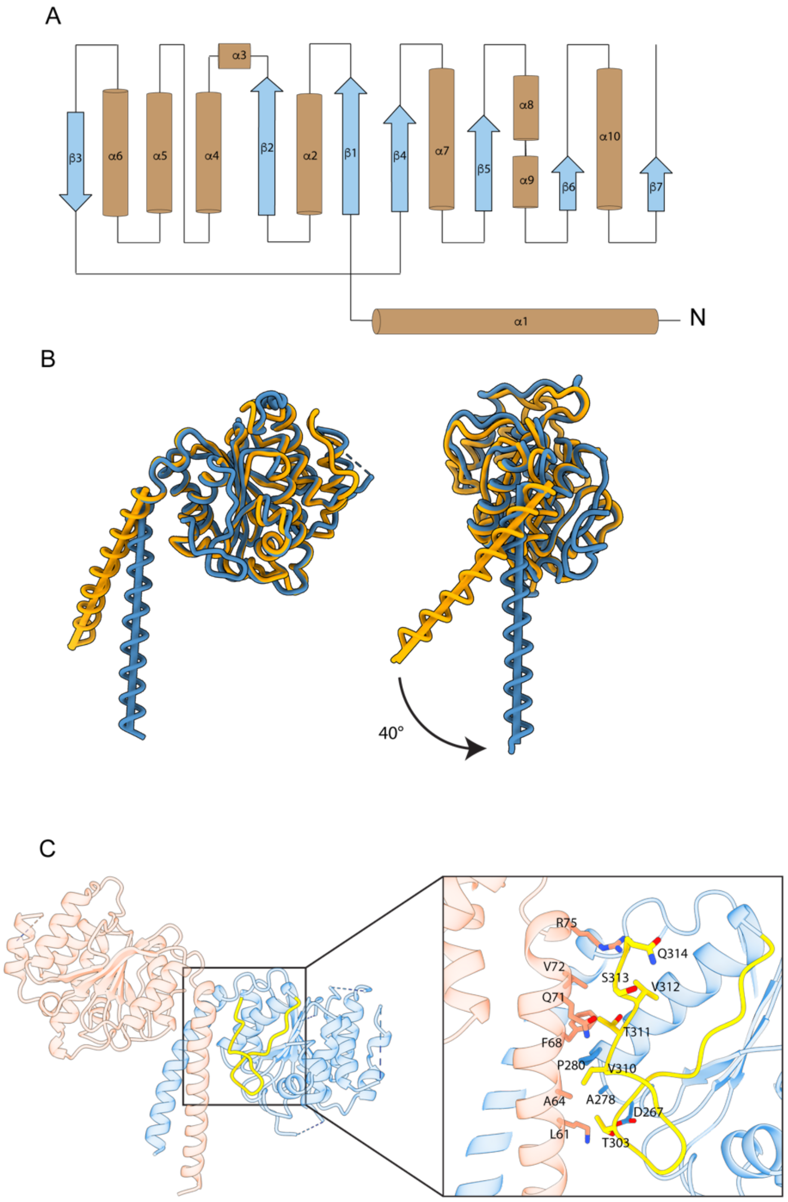

Figure S1. Structure of *Mt*SteB. (A) The secondary structure topology of *Mt*SteB partially resembles the $(\beta/\alpha)_5$ topology of receiver domains from bacterial response regulators. **(B)** Despite a rather low sequence identity (30%), the structural cores of *Mt*SteB (in blue) and *Cg*SteB (in orange) are almost identical. The major differences are observed in the region connecting the C-terminal globular core with the N-terminal helix, leading to a marked change in the orientation of the major helix axis. (**C**) Close up view of the *Mt*SteB dimerization interface between the helix from one protomer with the protein C-terminus from the other. All residues involved in the interface are shown as sticks and labelled. The C-terminal region shown in yellow is missing in the construct of monomeric *Cg*SteB (1) and might explain why this protein crystallized as a monomer.

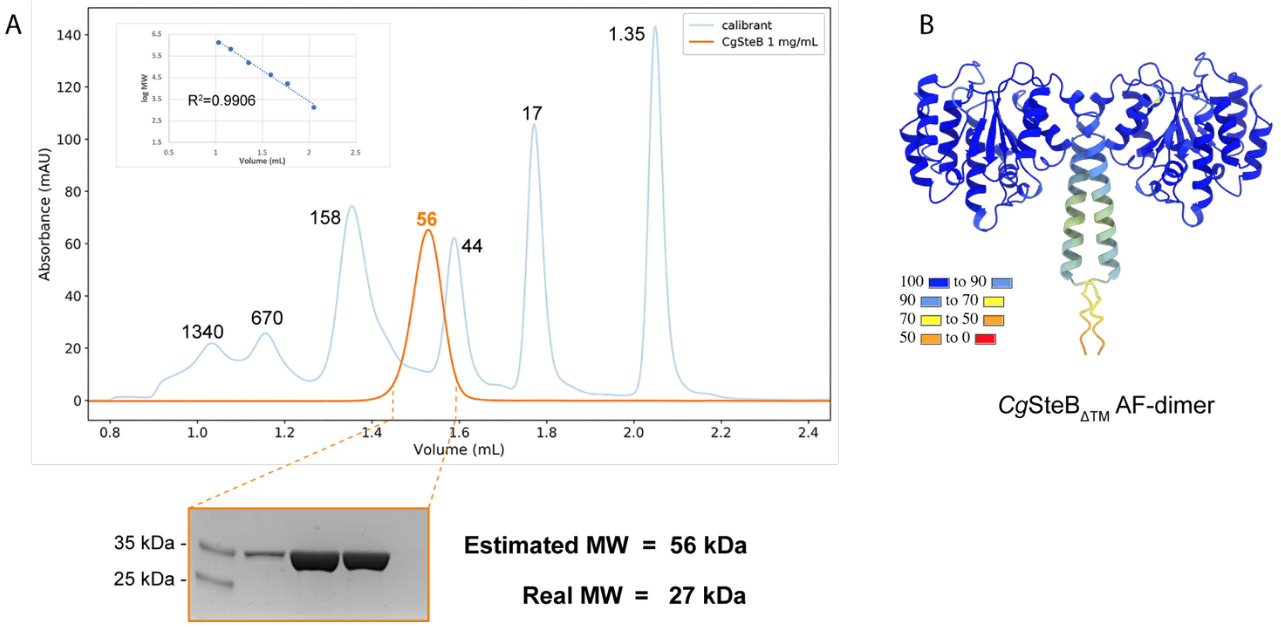

Figure S2. Oligomerization state of *Cg*SteB$_{\Delta TM}$. (**A**) The upper panel shows the Superdex 200 3.2/300 analytical size exclusion chromatography (SEC). The *Cg*SteB$_{\Delta TM}$ (residue range 36-295) profile is shown in orange, the calibrant profile is shown in blue and the molecular weight (kDa) is reported above each peak. The calibration curve, shown as inset, is based on Thyroglobulin (elution volume=1.03 and 1.16 mL, for the dimer and monomer, respectively), γ-globulin (elution volume=1.35 mL), Ovalbumin (1.59 mL), Myoglobin (1.77 mL), Vitamin B12 (2.05 mL). The lower panel shows the SDS-PAGE gel for the equivalent *Cg*SteB$_{\Delta TM}$ fractions and MW estimation. (**B**) AF prediction for the *Cg*SteB$_{\Delta TM}$ homodimer (pLDDT=95.2, ipTM+pTM=0.85).

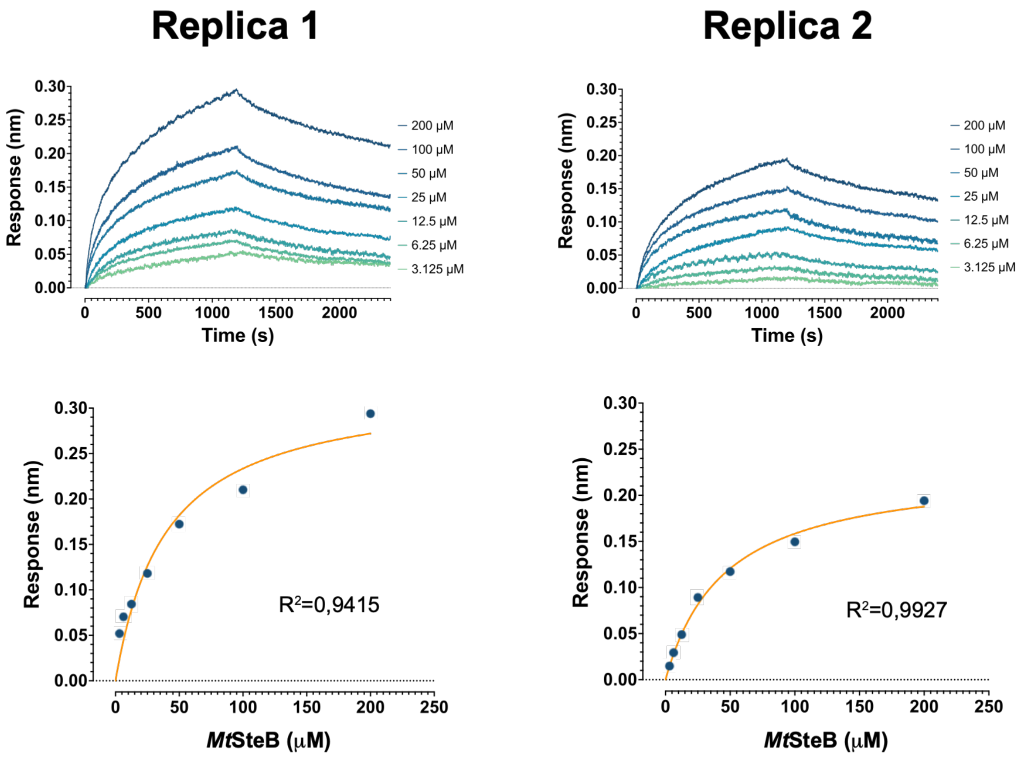

Figure S3. Two independent BLI experiments showing the interaction profiles for the *Mt*RipA-*Mt*SteB interaction used to calculate the dissociation constant shown in Fig. 1D.

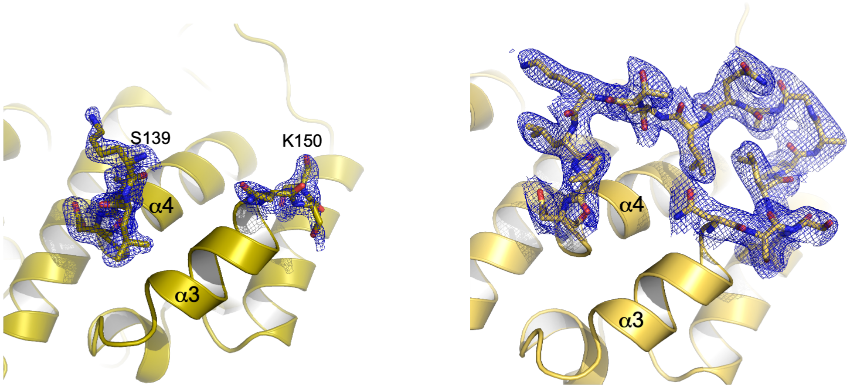

Figure S4. Close view of the α3-α4 loop in apo (left panel) and holo (right panel) *Mt*SteB, with the corresponding electron densities contoured at 1 σ.

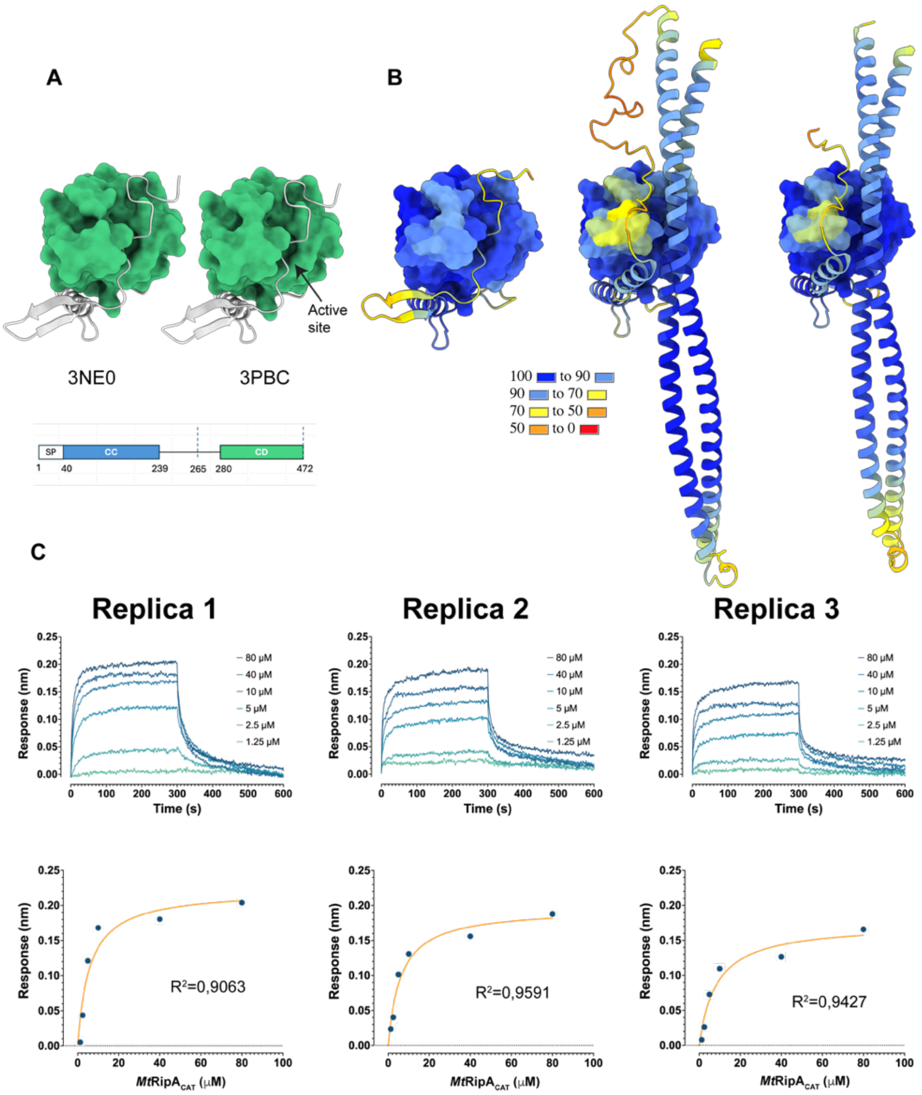

Figure S5. **The *Mt*RipA catalytic domain is autoinhibited by the N-terminal CC. (A)** Available crystal structures of truncated forms of the *Mt*RipA protein (residues 265-472), showing the NlpC/P60 catalytic domain (residues 280-472, green molecular surface) occluded by the N-terminal segment 264-279 (grey cartoon). The PDB codes and the modular organization of *Mt*RipA (SP: signal peptide, CC: coiled-coil domain, CD: catalytic domain) are shown below, with the limits of the truncated protein indicated by dotted lines. **(B)** The AF-predicted models of three different *Mt*RipA constructs color-coded by model confidence (left panel: construct comprising residues 265-472, pLDDT=91.5; central panel: construct comprising residues 40-472, pLDDT=86.6; and right panel: construct comprising residues 40-239+265-472, pLDDT=91.4, ipTM+pTM=0.88). These predictions revealed that the N-terminal CC, when present, binds to the active site. In all cases, the catalytic core (residues 280-472), oriented as in panel A, is shown in molecular surface representation and the different N-terminal extensions in cartoon representation. Color keys for AF pLDDT residue scoring are also shown. **(C)** Three independent BLI experiments showing the interaction profiles for the *Mt*RipA-*Mt*RipA$_{CAT}$ interaction used to calculate the dissociation constant shown in Fig. 1F.

A

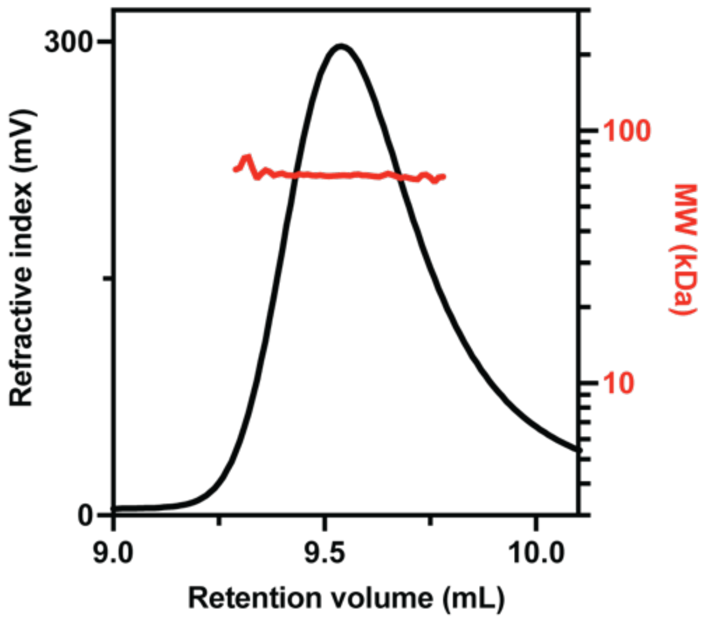

B

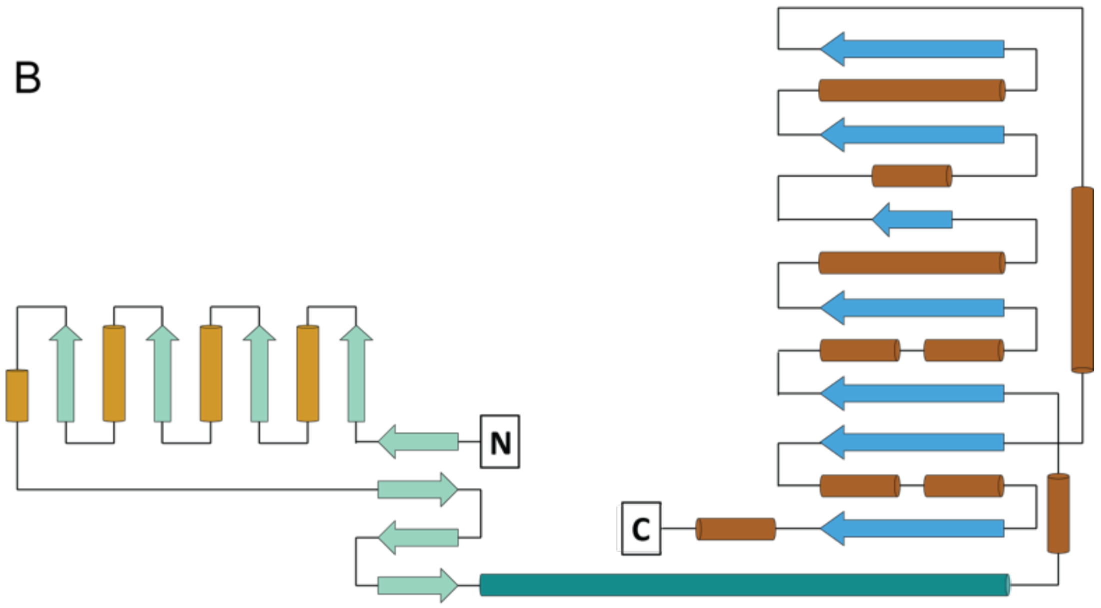

Figure S6. **(A)** SEC-SLS experiments show that *Mt*SteA elutes as a single peak (black curve) with an apparent MW of 66 kDa (red curve), close to the predicted MW of the dimer (70 kDa). **(B)** *Mt*SteA secondary structure diagram, colored by structural domains (light brown/green: N-terminal domain, dark green: central helix, dark brown/blue: C-terminal domain).

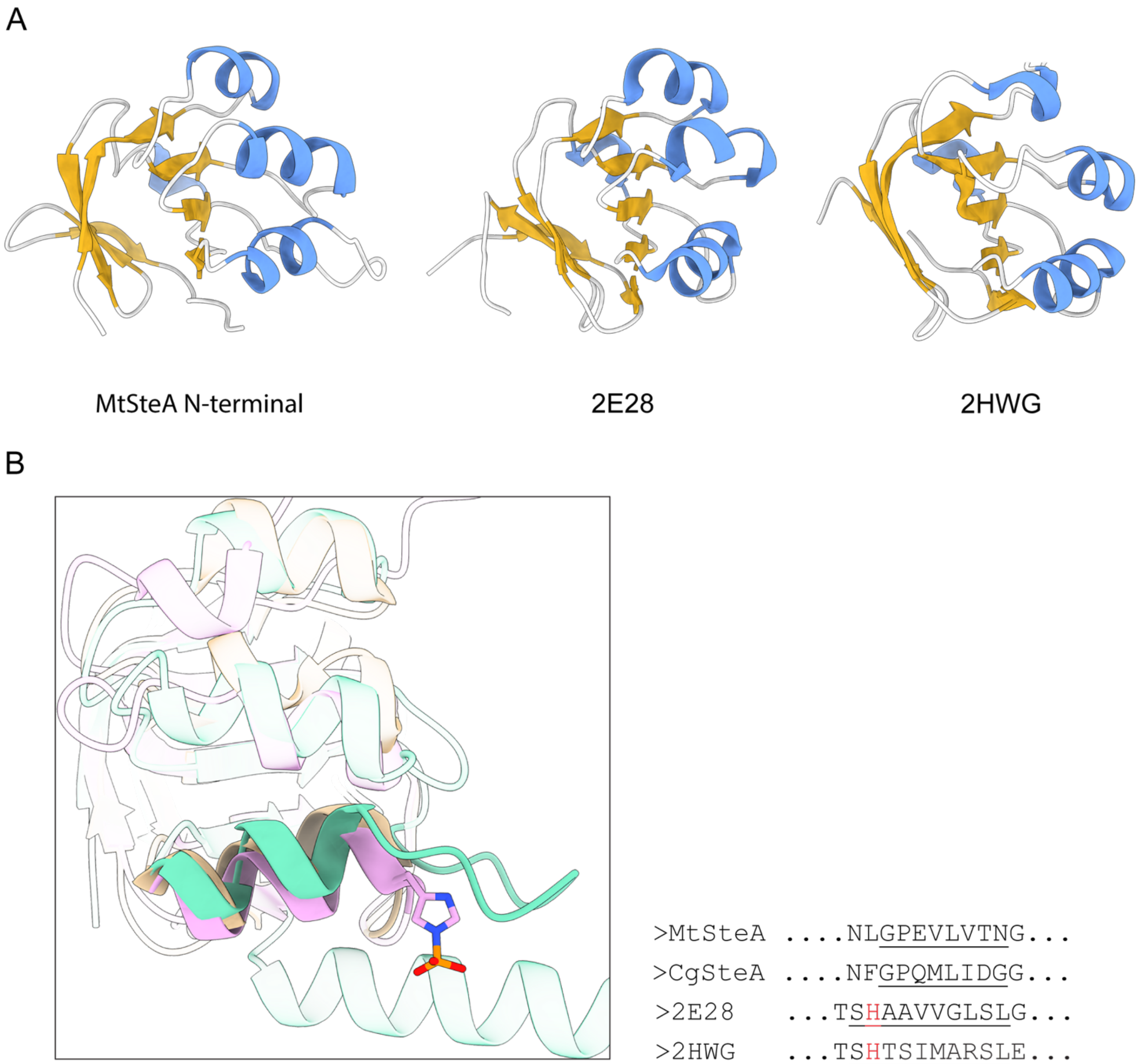

Figure S7. Structural similarity of the SteA N-terminal domain. (**A**) The N-terminal domain (left) can be superimposed with the phosphohistidine domains of pyruvate kinase (PDB code 2E28, (2)) with a rmsd of 1.1 Å for 48 equivalent Cα positions, and the phosphoenoylpyruvate:sugar phosphotransferase system (PDB code 2HWG, (3)) with a rmsd of 1.0 Å for 25 equivalent Cα positions. Structures are colored according to secondary structure (**B**) Close view of superposed SteA (green), 2E28 (tan) and 2HWG (pink). The (phospho)histidine residue is missing in *Mt*SteA. The partial sequence alignment of this region is shown at right.

A

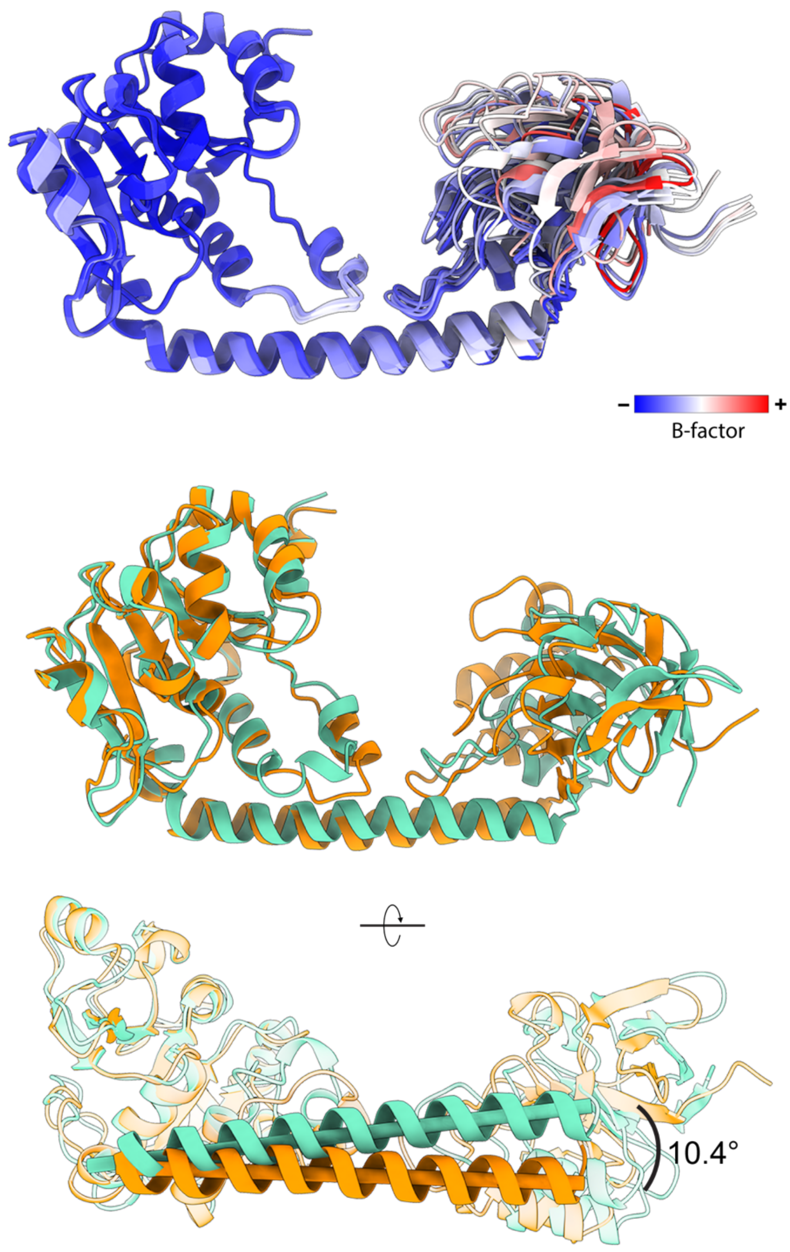

Figure S8. **Structural flexibility of the SteA N-terminal domain. (A)** Superposition of the 8 crystallographically independent molecules of *Cg*SteA color-coded according to B values. The highest B values are found for the N-terminal domain, on the right. **(B)** Two different views of the superposition between *Mt*SteA (green) and the least similar *Cg*SteA molecule (orange).

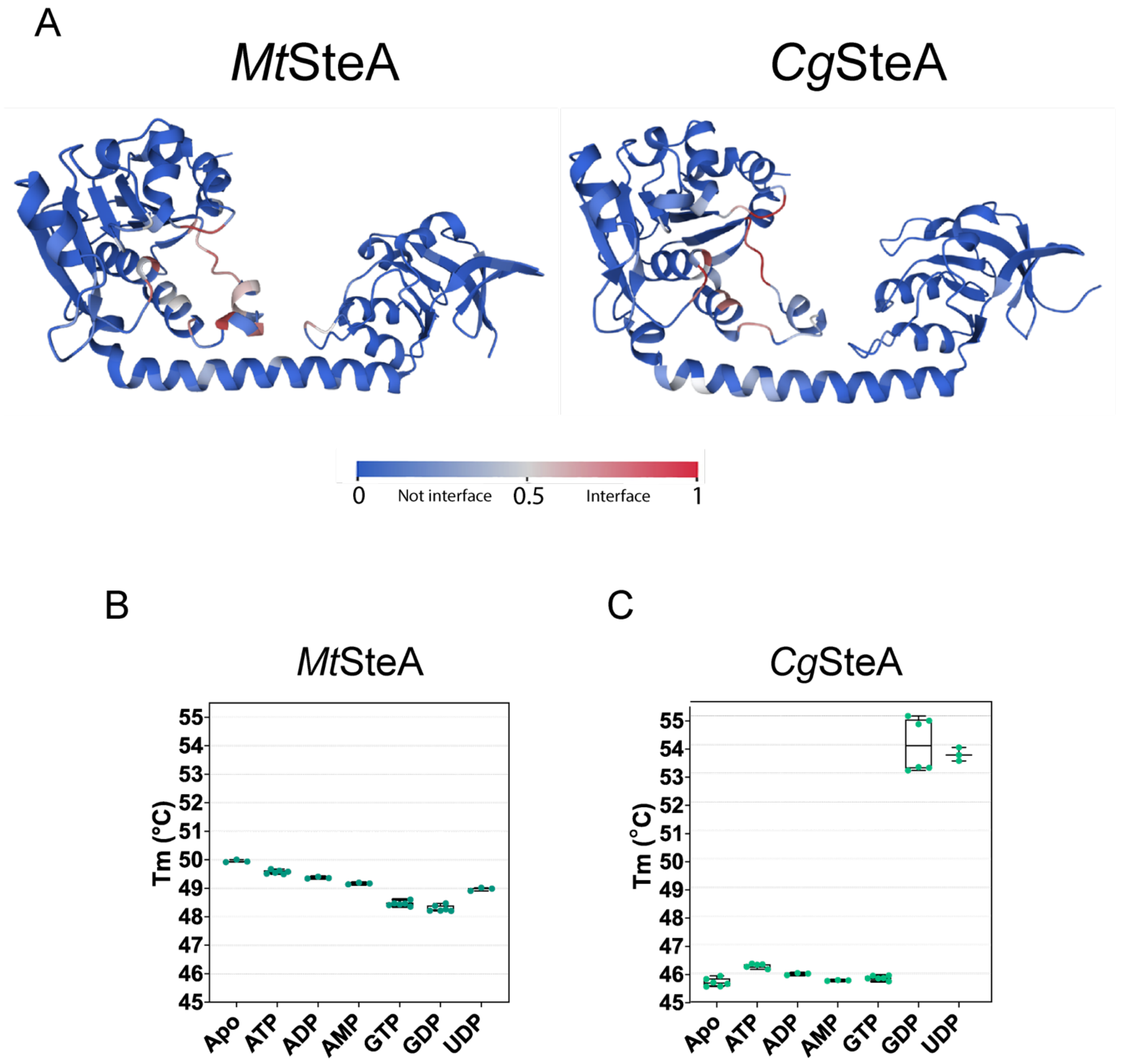

Figure S9. **The conserved ligand-binding pocket of SteA.** (**A**) The conserved pockets of *Mt*SteA (left) and *Cg*SteA (right) are both predicted to bind carbohydrate using a deep learning approach trained on protein-carbohydrate interfaces. (**B, C**) Binding of different phosphonucleotides to *Mt*SteA (**B**) and *Cg*SteA (**C**) as assessed by differential scanning fluorimetry (nanoDSF). No binding could be detected for *Mt*SteA in the presence or absence of 8 mM Mg^{2+}. However, the results indicated that *Cg*SteA can specifically bind UDP or GDP.

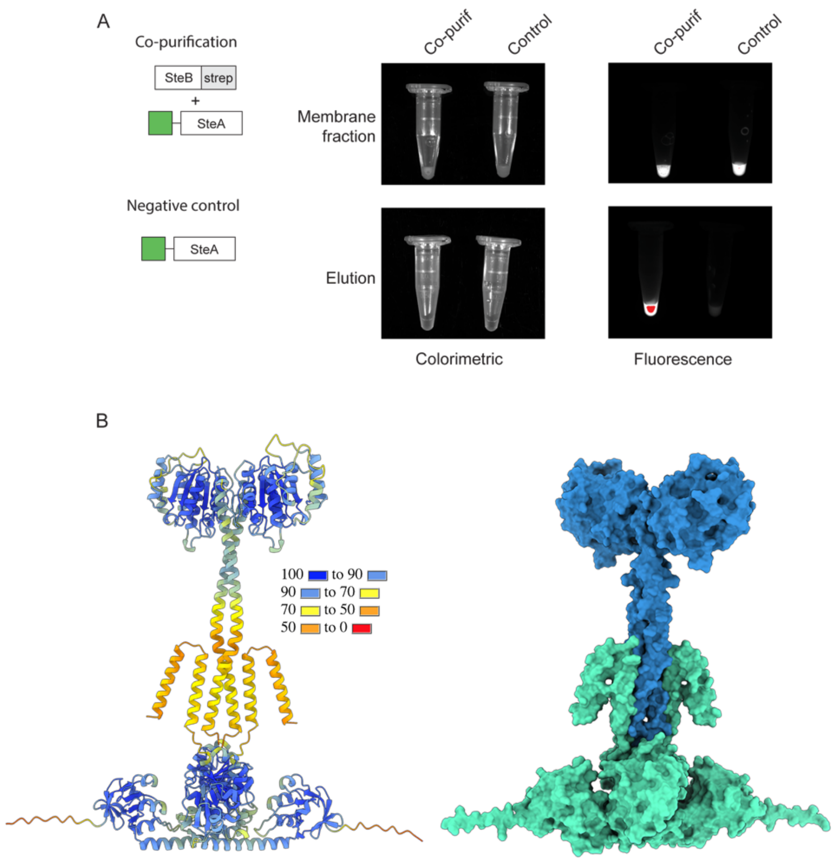

Figure S10. ***Mt*SteA and *Mt*SteB form a stable transmembrane physical complex.** (**A**) A schematic view of the tagged constructs used for co-expression and purification of the two proteins is shown at the left, with the mNeon fusion depicted as a green square. A strain expressing only mNeon-SteA is used as negative control. The right panel shows the membrane fraction (50 uL) and the elution fraction from the Strep-Tactin resin (bottom row) imaged in a ChemiDoc system by colorimetric or fluorescence (Alexa 488) detection. The membrane fractions for the co-purified proteins and the negative control show comparable fluorescence signal. In contrast, the fluorescence signal in the elution fractions is only retained in the tube corresponding to the co-purified proteins (note the saturated pixels), whereas it is negligible in the negative control. This data indicates that mNeon-SteA is retained on the resin only when co-expressed with SteB-Strep. See also Fig. 4A. (**B**) AlphaFold-predicted model of the heterotetramer (*Mt*SteA/*Mt*SteB)$_2$ color-coded according to model confidence (left panel, plDDT=84, pTM+ipTM=0.46) or protein organization (right panel), with the *Mt*SteA dimer shown in green and the *Mt*SteB dimer in blue. Color keys for AF pLDDT residue scoring are shown for the left panel.

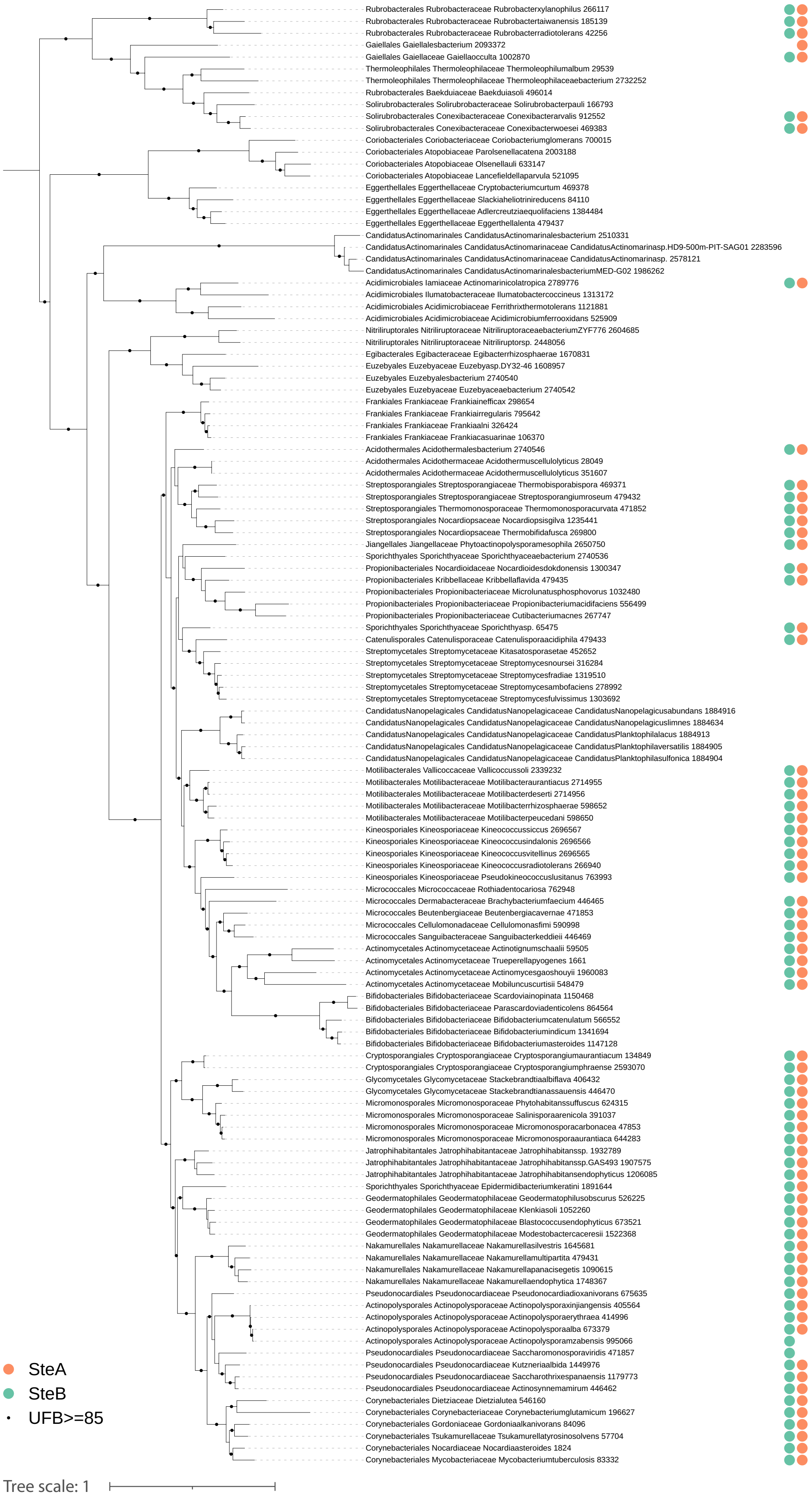
Rubrobacterales Rubrobacteraceae Rubrobacterxylanophilus 266117
Rubrobacterales Rubrobacteraceae Rubrobactertaiwanensis 185139
Rubrobacterales Rubrobacteraceae Rubrobacterradiotolerans 42256
Gaiellales Gaiellalesbacterium 2093372
Gaiellales Gaiellaceae Gaiellaocculta 1002870
Thermoleophilales Thermoleophilaceae Thermoleophilumalbum 29539
Thermoleophilales Thermoleophilaceae Thermoleophilaceaebacterium 2732252
Rubrobacterales Baekduiaceae Baekduiasoli 496014
Solirubrobacterales Solirubrobacteraceae Solirubrobacterpauli 166793
Solirubrobacterales Conexibacteraceae Conexibacterarvalis 912552
Solirubrobacterales Conexibacteraceae Conexibacterwoesei 469383
Coriobacteriales Coriobacteriaceae Coriobacteriumglomerans 700015
Coriobacteriales Atopobiaceae Parolsenellacatena 2003188
Coriobacteriales Atopobiaceae Olsenellauli 633147
Coriobacteriales Atopobiaceae Lancefieldellaparvula 521095
Eggerthellales Eggerthellaceae Cryptobacteriumcurtum 469378
Eggerthellales Eggerthellaceae Slackiaheliotrinireducens 84110
Eggerthellales Eggerthellaceae Adlercreutziaequolifaciens 1384484
Eggerthellales Eggerthellaceae Eggerthellalenta 479437
CandidatusActinomarinales CandidatusActinomarinalesbacterium 2510331
CandidatusActinomarinales CandidatusActinomarinaceae CandidatusActinomarinasp.HD9-500m-PIT-SAG01 2283596
CandidatusActinomarinales CandidatusActinomarinaceae CandidatusActinomarinasp. 2578121
CandidatusActinomarinales CandidatusActinomarinalesbacteriumMED-G02 1986262
Acidimicrobiales Iamiaceae Actinomarinicolatropica 2789776
Acidimicrobiales Ilumatobacteraceae Ilumatobactercoccineus 1313172
Acidimicrobiales Acidimicrobiaceae Ferrithrixthermotolerans 1121881
Acidimicrobiales Acidimicrobiaceae Acidimicrobiumferrooxidans 525909
Nitriliruptorales Nitriliruptoraceae NitriliruptoraceaebacteriumZYF776 2604685
Nitriliruptorales Nitriliruptoraceae Nitriliruptorsp. 2448056
Egibacterales Egibacteraceae Egibacterrhizosphaerae 1670831
Euzebyales Euzebyaceae Euzebyasp.DY32-46 1608957
Euzebyales Euzebyalesbacterium 2740540
Euzebyales Euzebyaceae Euzebyaceaebacterium 2740542
Frankiales Frankiaceae Frankiainefficax 298654
Frankiales Frankiaceae Frankiairregularis 795642
Frankiales Frankiaceae Frankiaalni 326424
Frankiales Frankiaceae Frankiacasuarinae 106370
Acidothermales Acidothermalesbacterium 2740546
Acidothermales Acidothermaceae Acidothermuscellulolyticus 28049
Acidothermales Acidothermaceae Acidothermuscellulolyticus 351607
Streptosporangiales Streptosporangiaceae Thermobisporabispora 469371
Streptosporangiales Streptosporangiaceae Streptosporangiumroseum 479432
Streptosporangiales Thermomonosporaceae Thermomonosporacurvata 471852
Streptosporangiales Nocardiopsaceae Nocardiopsisgilva 1235441
Streptosporangiales Nocardiopsaceae Thermobifidafusca 269800
Jiangellales Jiangellaceae Phytoactinopolysporamesophila 2650750
Sporichthyales Sporichthyaceae Sporichthyaceaebacterium 2740536
Propionibacteriales Nocardioidaceae Nocardioidesdokdonensis 1300347
Propionibacteriales Kribbellaceae Kribbellaflavida 479435
Propionibacteriales Propionibacteriaceae Microlunatusphosphovorus 1032480
Propionibacteriales Propionibacteriaceae Propionibacteriumacidifaciens 556499
Propionibacteriales Propionibacteriaceae Cutibacteriumacnes 267747
Sporichthyales Sporichthyaceae Sporichthyasp. 65475
Catenulisporales Catenulisporaceae Catenulisporaacidiphila 479433
Streptomycetales Streptomycetaceae Kitasatosporasetae 452652
Streptomycetales Streptomycetaceae Streptomycesnoursei 316284
Streptomycetales Streptomycetaceae Streptomycesfradiae 1319510
Streptomycetales Streptomycetaceae Streptomycesambofaciens 278992
Streptomycetales Streptomycetaceae Streptomycesfulvissimus 1303692
CandidatusNanopelagicales CandidatusNanopelagicaceae CandidatusNanopelagicusabundans 1884916
CandidatusNanopelagicales CandidatusNanopelagicaceae CandidatusNanopelagicuslimnes 1884634
CandidatusNanopelagicales CandidatusNanopelagicaceae CandidatusPlanktophilalacus 1884913
CandidatusNanopelagicales CandidatusNanopelagicaceae CandidatusPlanktophilaversatilis 1884905
CandidatusNanopelagicales CandidatusNanopelagicaceae CandidatusPlanktophilasulfonica 1884904
Motilibacterales Vallicoccaceae Vallicoccussoli 2339232
Motilibacterales Motilibacteraceae Motilibacteraurantiacus 2714955
Motilibacterales Motilibacteraceae Motilibacterdeserti 2714956
Motilibacterales Motilibacteraceae Motilibacterrhizosphaerae 598652
Motilibacterales Motilibacteraceae Motilibacterpeucedani 598650
Kineosporiales Kineosporiaceae Kineococcussiccus 2696567
Kineosporiales Kineosporiaceae Kineococcusindalonis 2696566
Kineosporiales Kineosporiaceae Kineococcusvitellinus 2696565
Kineosporiales Kineosporiaceae Kineococcusradiotolerans 266940
Kineosporiales Kineosporiaceae Pseudokineococcuslusitanus 763993
Micrococcales Micrococcaceae Rothiadentocariosa 762948
Micrococcales Dermabacteraceae Brachybacteriumfaecium 446465
Micrococcales Beutenbergiaceae Beutenbergiacavernae 471853
Micrococcales Cellulomonadaceae Cellulomonasfimi 590998
Micrococcales Sanguibacteraceae Sanguibacterkeddieii 446469
Actinomycetales Actinomycetaceae Actinotignumschaalii 59505
Actinomycetales Actinomycetaceae Trueperellapyogenes 1661
Actinomycetales Actinomycetaceae Actinomycesgaoshouyii 1960083
Actinomycetales Actinomycetaceae Mobiluncuscurtisii 548479
Bifidobacteriales Bifidobacteriaceae Scardoviainopinata 1150468
Bifidobacteriales Bifidobacteriaceae Parascardoviadenticolens 864564
Bifidobacteriales Bifidobacteriaceae Bifidobacteriumcatenulatum 566552
Bifidobacteriales Bifidobacteriaceae Bifidobacteriumindicum 1341694
Bifidobacteriales Bifidobacteriaceae Bifidobacteriumasteroides 1147128
Cryptosporangiales Cryptosporangiaceae Cryptosporangiumaurantiacum 134849
Cryptosporangiales Cryptosporangiaceae Cryptosporangiumphraense 2593070
Glycomycetales Glycomycetaceae Stackebrandtiaalbiflava 406432
Glycomycetales Glycomycetaceae Stackebrandtianassauensis 446470
Micromonosporales Micromonosporaceae Phytohabitanssuffuscus 624315
Micromonosporales Micromonosporaceae Salinisporaarenicola 391037
Micromonosporales Micromonosporaceae Micromonosporacarbonacea 47853
Micromonosporales Micromonosporaceae Micromonosporaaurantiaca 644283
Jatrophihabitantales Jatrophihabitantaceae Jatrophihabitanssp. 1932789
Jatrophihabitantales Jatrophihabitantaceae Jatrophihabitanssp.GAS493 1907575
Jatrophihabitantales Jatrophihabitantaceae Jatrophihabitansendophyticus 1206085
Sporichthyales Sporichthyaceae Epidermidibacteriumkeratini 1891644
Geodermatophilales Geodermatophilaceae Geodermatophilusobscurus 526225
Geodermatophilales Geodermatophilaceae Klenkiasoli 1052260
Geodermatophilales Geodermatophilaceae Blastococcusendophyticus 673521
Geodermatophilales Geodermatophilaceae Modestobactercaceresii 1522368
Nakamurellales Nakamurellaceae Nakamurellasilvestris 1645681
Nakamurellales Nakamurellaceae Nakamurellamultipartita 479431
Nakamurellales Nakamurellaceae Nakamurellapanacisegetis 1090615
Nakamurellales Nakamurellaceae Nakamurellaendophytica 1748367
Pseudonocardiales Pseudonocardiaceae Pseudonocardiadioxanivorans 675635
Actinopolysporales Actinopolysporaceae Actinopolysporaxinjiangensis 405564
Actinopolysporales Actinopolysporaceae Actinopolysporaerythraea 414996
Actinopolysporales Actinopolysporaceae Actinopolysporaalba 673379
Actinopolysporales Actinopolysporaceae Actinopolysporamzabensis 995066
Pseudonocardiales Pseudonocardiaceae Saccharomonosporaviridis 471857
Pseudonocardiales Pseudonocardiaceae Kutzneriaalbida 1449976
Pseudonocardiales Pseudonocardiaceae Saccharothrixespanaensis 1179773
Pseudonocardiales Pseudonocardiaceae Actinosynnemamirum 446462
Corynebacteriales Dietziaceae Dietzialutea 546160
Corynebacteriales Corynebacteriaceae Corynebacteriumglutamicum 196627
Corynebacteriales Gordoniaceae Gordoniaalkanivorans 84096
Corynebacteriales Tsukamurellaceae Tsukamurellatyrosinosolvens 57704
Corynebacteriales Nocardiaceae Nocardiaasteroides 1824
Corynebacteriales Mycobacteriaceae Mycobacteriumtuberculosis 83332
SteA
SteB
UFB>=85
Tree scale: 1

Figure S11. Distribution of proteins SteA and SteB in the tree of Actinobacteria. Phylogeny of representative Actinobacteria with SteA/SteB presence-absence mapped. Labels indicate order, family, species, and TaxID. Colored dots indicate detection of SteA and SteB according to the color key. Black dots indicate branches with UFB (ultrafast bootstrap) support ≥ 85. The scale bar indicates substitutions per site.

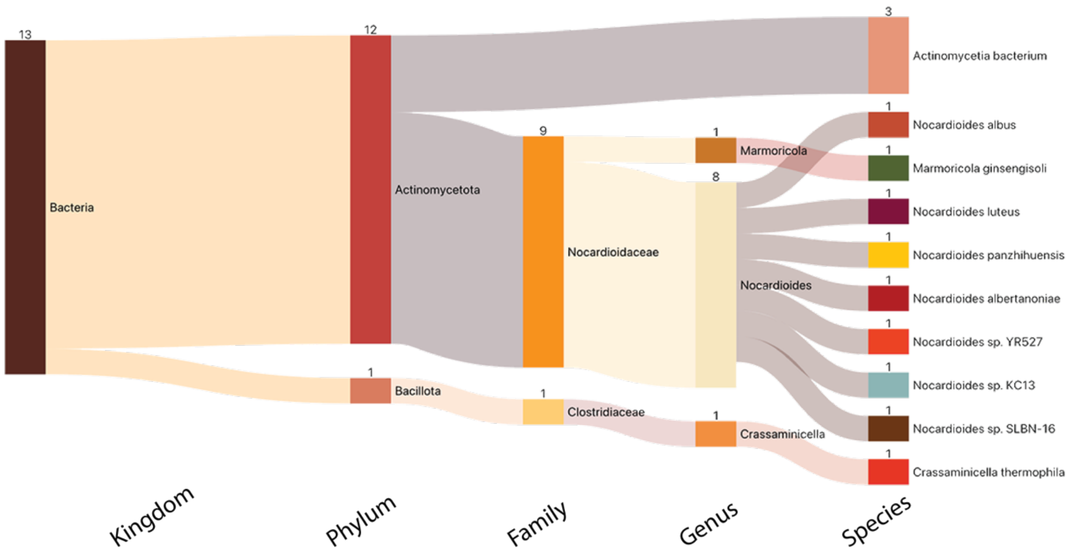

B

AFDB accession	Species	Sequence length	Average pLDDT
AF-A0A7K1ISP0-F1	*Actinobacteria bacterium*	634	76.31
AF-A0A7K1IT68-F1	*Actinobacteria bacterium*	631	62.34
AF-A0A6I2W638-F1	*Actinobacteria bacterium*	631	64.4
AF-A0A7W5A964-F1	*Nocardioides albus*	643	81.31
AF-A0A3N0CJS8-F1	*Marmoricola ginsengisoli*	627	83.88
AF-A0A1J4N9N2-F1	*Nocardioides luteus*	605	81.62
AF-A0A7Z0IRV9-F1	*Nocardioides panzhihuensis*	649	81.31
AF-A0A543A801-F1	*Nocardioides albertanoniae*	634	81.5
AF-A0A1G8XB47-F1	*Nocardioides sp. YR527*	651	81.69
AF-A0A5C0SEP3-F1	Nocardioides sp. KC13	643	82.44
AF-A0A542TAP4-F1	*Nocardioides sp. SLBN-16*	641	81.62
AF-A0A5C0SEP3-F1	*Crassaminicella thermophila*	675	83.44
AF-A0A6J7E7J6-F1	*freshwater metagenome*	634	77.25

C

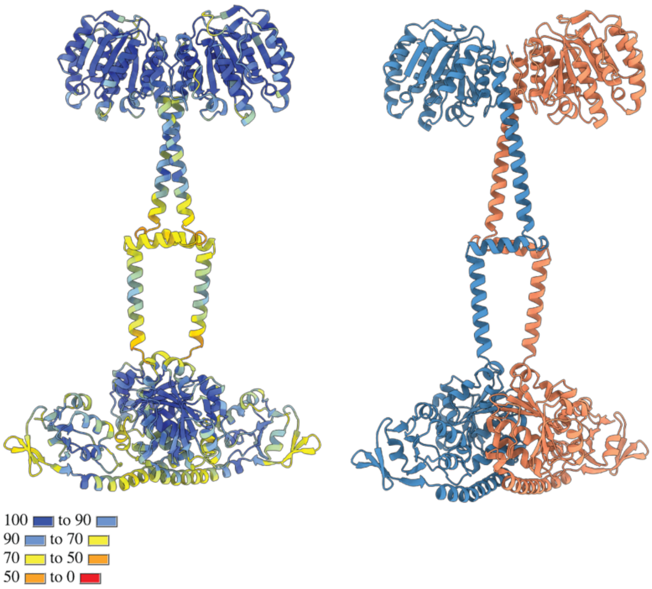

Figure S12. Gene fusion of the SteAB module across bacteria in the FoldSeek-based AlphaFold Data Base (AFDB) clusters. (**A**) Taxonomic distribution of *steAB* fused genes from the AFDB, obtained using iterative FoldSeek searches (4). (**B**) Summary of the 13 hits. (**C**) AF dimer prediction for the representative member with the highest pLDDT score, from *M. ginsengisoli* (pLDDT=83.88, ipTM+pTM=0.48). The structures are colored according to pLDDT (left) or by polypeptide chain (right). The overall organization resembles that of the SteAB complex, although the relatively low pLDDT values for the TM helices indicate that the actual structure of this region may differ from that shown here.

A

B

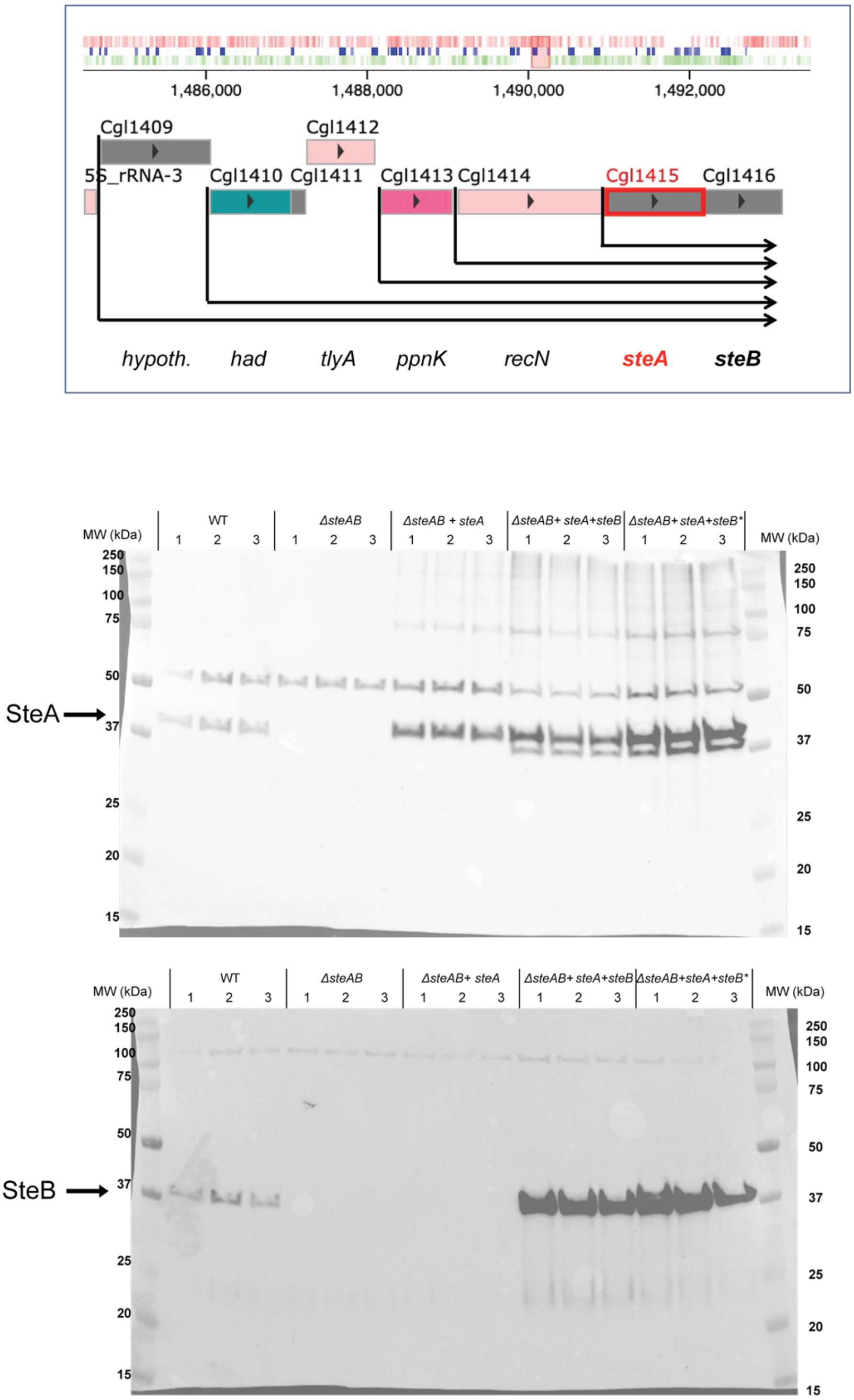

Figure S13. (A) Transcription start sites as described in (5) **(B)** Western Blots anti-SteA (top) and anti-SteB (bottom) of whole cell extracts (60 µg) from the different strains shown in Fig. 5. An arrow indicates the specific signal for *Cg*SteA and *Cg*SteB, respectively. Non-specific bands in each blot can be used as a loading control.

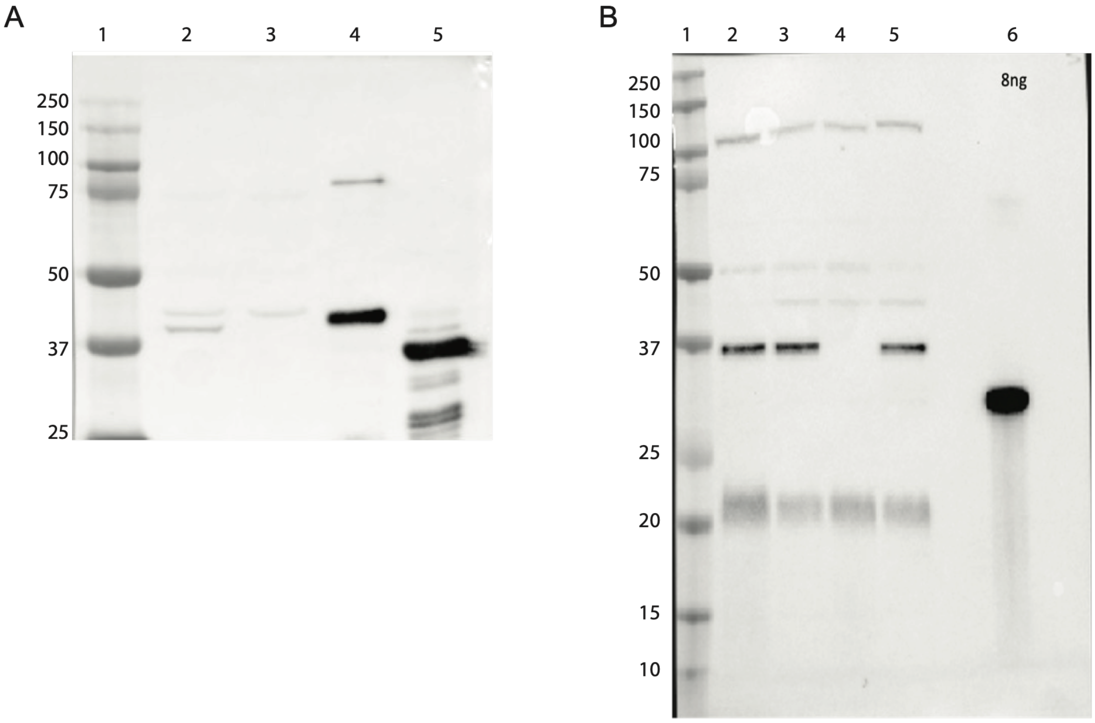

Figure S14. Antibody characterization. (**A**) Western Blot using purified anti-SteA antibody. Lane 1: molecular weight markers [kDa]; lane 2: total cell extract (120 µg) of *Cglu*; lane 3: total cell extract (120 µg) of *Cglu_ΔsteAB*; lane 4: recombinant full-length His-SteA (0.1 µg); lane 5: recombinant soluble His-SteA (0.4 µg) (**B**) Western Blot using purified anti-SteB antibody. Lane 1: molecular weight markers, kDa; lane 2: total cell extract (120 µg) of *Cglu*; lane 3: total cell extract (120 µg) of *Cglu_empty plasmid*; lane 4: total cell extract (120 µg) of *Cglu_ΔsteAB*; lane 5: total cell extract (120 µg) of *Cglu_ΔsteAB* + *Scarlet-SteB* (note that in these conditions Scarlet-SteB is not expressed); lane 6: recombinant soluble His-SteB (8 ng).

Supplementary Table S1. Crystallographic data collection and refinement statistics.

Crystal	*Mt*SteA$	*Cg*SteA$	*Cg*SteA$ SeMet-labeled	*Mt*SteB	*Mt*SteB phasing	*Mt*SteB-RipA$_{CC}$$
**Data Collection**	2023/09/09	2023/12/16	2020/06/23	2022/09/09	2022/09/09	2022/11/15
Synchrotron Beamline	Soleil Proxima 2A	Soleil Proxima 2A	Soleil Proxima 2A	Soleil Proxima 2A	Soleil Proxima 2A	ESRF ID23-1
Wavelength (Å)	0.98011	0.97934	0.97918	1.07169	1.54981	1.59281
Space group	C2221	P212121	P212121	C2221	C2	C2
Unit cell a, b, c (Å, °)	72.3, 79.8, 132.4<br>90.0 90.0 90.0	126.5 128.7 211.8<br>90.0 90.0 90.0	123.7 127.5 212.0<br>90.0 90.0 90.0	64.4 162.3 148.8<br>90.0 90.0 90.0	164.0 66.2 155.9<br>90.0 91.6 90.0	79.8 225.1 70.3<br>90.0 124.4 90.0
Resolution (Å)	66.187 - 2.167<br>(2.43 - 2.167)*	105.925 - 2.050<br>(2.333 - 2.050)*	109.230 - 2.522<br>(2.784 - 2.522)*	81.173 -2.035<br>(2.071 - 2.035)*	81.973 - 2.403<br>(2.445 - 2.403)*	63.020 - 2.217<br>(2.401 - 2.217)*
*R*pim	0.046 (0.580)	0.044 (0.562)	0.072 (0.675)	0.048 (0.390)	0.028 (0.306)	0.046 (0.506)
I / s(I)	9.6 (1.6)	9.2 (1.8)	9.8 (1.6)	10.5 (2.2)	16 (2.2)	9.4 (1.5)
Completeness (%)	91.6 (78.1)	95.6 (73.9)	95.4 (69.5)	99.3 (97.1)	100 (100)	88.5 (51.0)
CC(1/2)	0.99 (0.62)	0.992 (0.703)	0.998 (0.695)	0.994 (0.657)	0.998 (0.808)	0.996 (0.664)
Multiplicity	14.8 (14.0)	13.6 (12.3)	13.4 (13.7)	9.0 (7.1)	13.3 (13.4)	5.2 (4.4)
Total observations	166454 (7860)	1723135 (78150)	1086805 (55663)	449914 (17167)	869212 (43594)	162070 (6879)
Unique observations	11250 (562)	126836 (6342)	81282 (4064)	50045 (2414)	65533 (3257)	31204 (1560)
**Refinement**&						
Resolution range (Å)	66.19 - 2.167	105.92 - 2.050		– 2.035		58.03 - 2.220
No. reflections	11250	126836		50044		31165
Rwork/ Rfree	0.2053 / 0.2597	0.2052 / 0.2382		0.2224 / 0.2470		0.2056 / 0.2372
**No. atoms**						
Protein	2457	19495		5562		3335
Ligands/ions	-	-		33 (Cd)<br>1 (ACT)<br>1 (PG4)		2 (HEZ)
Solvent	117	1307		324		291
**Average B-factors (Å^2)**						
Protein	59.70	64.04		40.49		54.69
Ligand/ions	-	-		40.60 (Cd)<br>50.53 (ACT)<br>55.44 (PG4)		54.46 (HEZ)
Solvent	47.57	55.76		45.05		48.15
**R.m.s deviations^**						
Bond lengths (Å)	0.01	0.01		0.01		0.01
Bond angles (°)	1.54	1.46		1.45		1.46
Ramachandran favored (%)	97.54	97.17		99.07		98.22
Ramachandran outliers (%)	0	0		0		0

$Data sets subjected to anisotropic resolution surface cut-off with STARANISO.

*Values in parenthesis correspond to the highest resolution shell.

& Refinement statistics are from Buster (version 20240710).

^Computed with MolProbity.

Supplementary Table S2. Plasmids and strains used in this study.

***E. coli* Strains**		
**Strain**	**Characteristics**	**Reference**
DH5*α*	F- endA1 Φ80dlacZΔM15 Δ(lacZYA-argF)U169 recA1 relA1 hsdR17(rK–mK+) deoR supE44 thi-1 gyrA96 phoA λ–; strain used for general cloning procedures	6
CopyCutter EPI400	F- mcrA Δ(mrr-hsdRMS-mcrBC) Φ80dlacZΔM15 ΔlacX74 recA1 endA1 araD139 Δ(ara, leu)7697 galU galK λ- rpsL (StrR) nupG trfA tonA pcnB4 dhfr	7
BL21(DE3)	F- ompT hsdSB(rB–mB–) gal dcm (DE3); host for protein production	8
C41(DE3)	F – ompT hsdSB (rB- mB-) gal dcm (DE3); host for protein production	9

***C. glutamicum* Strains**		
**Strain**	**Characteristics**	**Reference**
ATCC 13032	Biotin-auxotrophic wild type	10
ΔSteA	SteAB Knock out strain	This work

**Plasmids for *C. glutamicum* knock out generation**		
**Plasmid**	**Description**	**Reference**
pK19mobsacB	KanaR; plasmid for allelic exchange in *C. glutamicum*; (pK18 oriVEc, sacB, lacZα)	11
pk19-ΔSteAB	KanaR; pK19mobsacB derivative for CgSteAB (Cg1603/Cg1604) chromosomic deletion	This work

**Plasmids for recombinant protein expression in *E. coli***		
**Plasmid**	**Description**	**Reference**
pET-SUMO	AmpR; pET derivate containing a N-terminal His-tag followed by a SUMO protease cleavage site	This work
pET-SUMO-*Mt* $SteB_{\Delta TM}$	AmpR; pET derivate for *M. tuberculosis* Rv1698 (38-314) recombinant expression containing a N-terminal His-tag followed by a SUMO protease cleavage site	This work
pTGR5-T7	KanR; pTGR5 derivate for recombinant expression of protein under the control of a T7 promoter, with/without a N-terminal His-tag	This work
pET-SUMO-*Mt* $RipA_{\Delta TM}$	AmpR; pET derivate for *M. tuberculosis* Rv1477 (40-472) recombinant expression containing a N-terminal His-tag followed by a SUMO protease cleavage site	This work
pET-SUMO-*Mt* $RipA_{CC}$	AmpR; pET derivate for *M. tuberculosis* Rv1477 (40-240) recombinant expression containing a N-terminal His-tag followed by a SUMO protease cleavage site	This work
pET-SUMO-*Mt* $RipA_{CAT}$	AmpR; pET derivate for *M. tuberculosis* Rv1477 (261-472) recombinant expression containing a N-terminal His-tag followed by a SUMO protease cleavage site	This work
pET-SUMO-*Mt* $SteA_{\Delta TM}$	AmpR; pET derivate for *M. tuberculosis* Rv1697 (13-341) recombinant expression containing a N-terminal His-tag followed by a SUMO protease cleavage site	This work
pET-SUMO-*Cg* $SteA_{\Delta TM}$	AmpR; pET derivate for *C. glutamicum* Cg1603 (12-344) recombinant expression containing a N-terminal His-tag followed by a SUMO protease cleavage site	This work
pTGR-T7-*Cg* SteA	KanR; pTGR5 derivate for recombinant expression of *Cg* 1603(1-397) under the control of their own T7 promoter	This work
pTGR-T7-*Cg* SteB	KanR; pTGR5 derivate for recombinant expression of His-*Cg* 1604(1-321) under the control of their own T7 promoter	This work
pTGR-T7-*Cg* SteAB	KanR; pTGR5 derivate for recombinant co-expression of *Cg* 1603(1-397) and His-*Cg* 1604(1-321), both under the control of their own T7 promoter	This work
pTGR-PgntK-Alfa-mNeon-MtSteA	KanR; pTGR5 derivate for recombinant expression of *MtSteA* containing a N-terminal Alfa-mNeonGreen tag, under the control of PgntK promoter	This work
pTGR-PgntK-MtbSteB-strep	KanR; pTGR5 derivate for recombinant expression of *MtSteB* containing a C-terminal Strep tag, under the control of PgntK promoter	This work
pTGR-PgntK-MtbSteB-strep/PgntK-Alfa-mNeon-MtSteA	KanR; pTGR5 derivate for recombinant co-expression of *MtSteA* containing a N-terminal Alfa-mNeonGreen tag and *MtSteB* containing a C-terminal Strep tag, both under the control of their own PgntK promoter	This work

**Plasmids for recombinant protein expression in *C. glutamicum*.**		
**Plasmid**	**Description**	**Reference**
pUMS_3	KanaR; pTGR5 derivative in which *Ptac* was exchanged by *PgntK* promoter to control the expression of the EGFP protein	12
pUMS_3-PgntK	KanaR; pTGR5 derivative containing *PgntK* promoter (empty plasmid)	12
pUMS3-*Cg* SteA	KanR; pTGR5 derivate for expression of *Cg* 1603(1-397) under the control of the *PgntK* promoter	This work
pUMS3-*Cg* SteB	KanR; pTGR5 derivate for expression of *Cg* 1604(1-321) under the control of the *PgntK* promoter	This work
pUMS-*Cg* $SteB_{L146R}$	KanR; pTGR5 derivate for expression of *Cg1604L146R (1-321)* under the control of the *PgntK* promoter	This work
pUMS3-*Cg* SteAB	KanR; pTGR5 derivate for co-expression of *Cg* 1603(1-397) and *Cg* 1604(1-321), each under the control of their own *PgntK* promoter	This work
pUMS-*Cg* $SteAB_{L146R}$	KanR; pTGR5 derivate for co-expression of *Cg* 1603 (1-397) and *Cg* 1604_{L146R} (1-321), each under the control of their own *PgntK* promoter	This work

Supplementary Table S3. Oligonucleotide primers used in this study.

Plasmid	Oligonucleotide	Sequence 5' → 3' and properties[a]
**Plasmids for *C. glutamicum* knock out generation**		
**Plasmid**	**Oligonucleotide**	**Sequence 5' → 3' and properties[a]**
pk19-ΔSteAB	OligoAS_p68	**TGAGCGGATAACAATT**TCAC
	OligoAS_p69	**CAATTCCACACAACA**TACG
	OligoAS_p279	**TGTTGTGTGGAATTG**AGGTTGCTTTGACCTCCATTG
	OligoAS_p280	**GTTGCCTTCCTATGTGGA**ACCAAAGTCTCAACC
	OligoAS_p281	**TCCACATAGGAAGGCAAC**ATGGCTAAAC
	OligoAS_p282	**AATTGTTATCCGCTCA**GACCTGGCCTGGCAAAATG
	OligoAS_p283	CGGCTTTGATGAATCCCAGG
	OligoAS_p284	GACCTACCGGCTAGTTCCTC
**Plasmids for recombinant protein expression in *E. coli***		
**Plasmid**	**Oligonucleotide**	**Sequence 5' → 3' and properties[a]**
pET-SUMO-*Mt*SteB$_{\Delta TM}$	QG_90	**TAAAAGGGCGAGCTCAGATC**CGG
	QG_91	**ACCACCAATCTGCTCACGAT**GTGC
	QG_189	**ATCGTGAGCAGATTGGTGGT**CGCAGTGAAAAACGTGACCTGTACA
	QG_190	**GATCTGAGCTCGCCCTTTTA**TTGGGACACCGTCACGCTCAT
pET-SUMO-*Mt*RipA$_{\Delta TM}$	QG_90	**TAAAAGGGCGAGCTCAGATC**CGG
	QG_91	**ACCACCAATCTGCTCACGAT**GTGC
	QG_191	**ATCGTGAGCAGATTGGTGGT**GACCCCCAAACAGATACTATAGCAGCT
	QG_192	**GATCTGAGCTCGCCCTTTTA**GTATTCAATGTAACGAACCACGTATGGG
pET-SUMO-*Mt*RipA$_{CC}$	QG_90	**TAAAAGGGCGAGCTCAGATC**CGG
	QG_91	**ACCACCAATCTGCTCACGAT**GTGC
	QG_191	**ATCGTGAGCAGATTGGTGGT**GACCCCCAAACAGATACTATAGCAGCT
	QG_193	**GATCTGAGCTCGCCCTTTTA**TTCGGAAGACCATGCAACCAAGC
pET-SUMO-*Mt*RipA$_{CAT}$	QG_90	**TAAAAGGGCGAGCTCAGATC**CGG
	QG_91	**ACCACCAATCTGCTCACGAT**GTGC
	QG_194	**ATCGTGAGCAGATTGGTGGT**CGCGCTTGGGAC
	QG_192	**GATCTGAGCTCGCCCTTTTA**GTATTCAATGTAACGAACCACGTATGGG
pET-SUMO-*Mt*SteA$_{\Delta TM}$	QG_90	**TAAAAGGGCGAGCTCAGATC**CGG
	QG_91	**ACCACCAATCTGCTCACGAT**GTGC
	GC_7_Fw	**ATCGTGAGCAGATTGGTGGT**CGTCCGGGTTTGATCGGTATCG
	GC_7_Rv	**GATCTGAGCTCGCCCTTTTA**GCGATACAGGGTTGCGACCGC
pET-SUMO-*Cg*SteA$_{\Delta TM}$	QG_90	**TAAAAGGGCGAGCTCAGATC**CGG
	QG_91	**ACCACCAATCTGCTCACGAT**GTGC
	QG_92	**ATCGTGAGCAGATTGGTGGT**GACCTGCCCGGCCTGCAAG
	QG_93	**GATCTGAGCTCGCCCTTTTA**GCGCACTGTGTAAAGACTTGCGATGAC
pTGR-T7-*Cg*SteA	QG_110	**TAATTAACCTAGGCTGCTGC**CAC
	QG_111	**CATGGTATATCTCCTTCTTAAA**GTTAAACAAAATTATTTC
	QG_147	**TTTAAGAAGGAGATATACC**ATGGGCTGCATGAGTCTGTTCAACC
	QG_129	**GCAGCAGCCTAGGTTA**ATTACTATTTGAACCAACCCTGGACTGTCAG
pTGR-T7-*Cg*SteB	QG_110	**TAATTAACCTAGGCTGCTGC**CAC
	QG_112	**CCCGTGATGATGATGGTGAT**GAC
	QG_149	**ATCACCATCATCATCACGG**GGCTAAACGACGTGGAAGAGGC
	QG_131	**GCAGCAGCCTAGGTTAATTA**CTACTGCGCTGGCGCTGC
**Plasmids for recombinant protein expression in *C. glutamicum.***		
**Plasmid**	**Oligonucleotide**	**Sequence 5' → 3' and properties[a]**
pUMS3-*Cg*SteA	QG_127	**ATGGTCTTATCC**TTTCTTTGGTGGCGT
	QG_156	**GGATAAGACCAT**ATGGGCTGCATGAGTCTGTTCAACC
pUMS3-CgSteB	QG_165	**AGAAAGGATAAGACCAT**ATGGCTAAACGACGTGGAAGAGG
	QG_166	**TACCTTAAGCGGCCGCTTTA**CTACTGCGCTGGCGCTGC
pUMS3-*Cg*SteB$_{L146R}$	QG_185	**CAGCGTTCTGAA**ACCCAACTGGATCCAGG
	QG_186	**TTCAGAACGCTG**AGCGCCTGCAGG
	[a]Overlaps for Gibson assembly are written in bold letters.	

Video V1. Flexibility of the SteA N-terminal domain. Morphing between the *Mt*SteA and *Cg*SteA crystal structures illustrating the intrinsic flexibility of the N-terminal domain.

SI References

1. Q. Gaday, *et al.*, FtsEX-independent control of RipA-mediated cell separation in *Corynebacteriales*. *Proc Natl Acad Sci USA* **119**, e2214599119 (2022).

2. A. Teplyakov, *et al.*, Structure of phosphorylated enzyme I, the phosphoenolpyruvate:sugar phosphotransferase system sugar translocation signal protein. *Proc Natl Acad Sci* **103**, 16218–16223 (2006).

3. K. Suzuki, S. Ito, A. Shimizu-Ibuka, H. Sakai, Crystal structure of pyruvate kinase from *Geobacillus stearothermophilus*. *J Biochem* **144**, 305–312 (2008).

4. M. van Kempen, S.S. Kim, C. Tumeschelt, M. Mirdita, J. lee, C.L.M. Gilchrist, J. Söding, M. Steinegger, Fast and accurate protein structure search with Foldseek. *Nat Biotechnol* **42**, 243-246 (2022).

5. K. Pfeifer-Sancar, A. Mentz, C. Rückert, J. Kalinowski, Comprehensive analysis of the *Corynebacterium glutamicum* transcriptome using an improved RNAseq technique. *BMC Genomics* **14**, 888–888 (2013).

6. D. Hanahan, Studies on transformation of *Escherichia coli* with plasmids. *J Mol Biol* **166**, 557-80 (1983)

7. D. Hasking. *Epicentre Forum* **11**, 6 (2004).

8. F.W. Studier, B.A. Moffatt, User of bacteriophage T7 RNA polymerase to direct selective high-level expression of cloned genes. *J Mol Biol* **189**, 113-30 (1986).

9. B. Miroux, J. E. Walker, Over-production of proteins in *Escherichia coli*: mutant hosts that allow synthesis of some membrane proteins and globular proteins at high levels. *J Mol Biol* **260**, 289–298 (1996).

10. S. Kinoshita, S. Udaka, M. Shimono, Studies on the amino acid fermentation. *J Gen Appl Microbiol* **3**, 193-205 (1957)

11. A. Schäfer, *et al.*, Small mobilizable multi-purpose cloning vectors derived from the *Escherichia coli* plasmids pK18 and pK19: selection of defined deletions in the chromosome of *Corynebacterium glutamicum*. *Genetics* **145**, 69–73 (1994).

12. M. Martinez, *et al.*, Eukaryotic-like gephyrin and cognate membrane receptor coordinate corynebacterial cell division and polar elongation. *Nat Microbiol* **8**, 1896–1910 (2023).
